# Idiopathic Scoliosis as a Conversion Reaction to Stress with the Neural Effect of a “Distorting Mirror”

**DOI:** 10.3390/life16020270

**Published:** 2026-02-04

**Authors:** Vladimir Rodkin, Mitkhat Gasanov, Inna Vasilieva, Yuliya Goncharuk, Natalia Skarzhinskaia, Nwosu Chizaram, Stanislav Rodkin

**Affiliations:** 1 Knot Polyclinic at Taganrog Station, Pl. Vosstaniya 1, Office 105, 347904 Taganrog, Russia; 2 Department of Hospital Therapy, Yaroslav-the-Wise Novgorod State University, 173003 Veliky Novgorod, Russia; 3 N.V. Sklifosovsky Institute of Clinical Medicine, Department of Polyclinic Therapy, Sechenov First Moscow State Medical University, 119435 Moscow, Russia; inniva77@mail.ru; 4 Department of Traumatology, Orthopedics and Disaster Surgery, Sechenov First Moscow State Medical University, 119435 Moscow, Russia; goncharuk_yu_r@staff.sechenov.ru; 5 Department of Internal Medicine No. 1, Rostov State Medical University, 344022 Rostov-on-Don, Russia; 6 Research Laboratory “Medical Digital Images Based on the Basic Model”, Department of Bioengineering, Institute of Living Systems, Don State Technical University, 344000 Rostov-on-Don, Russia

**Keywords:** adolescent idiopathic scoliosis, brain lateralization, right-hemisphere dysfunction, conversion reaction, coping strategy, “distorting mirror effect”, gender differences, risk factors, prevention and treatment

## Abstract

**Objective:** To synthesize current evidence on the relationships between adolescent idiopathic scoliosis (AIS), stress-related mechanisms, neuroanatomical asymmetry, and mental disorders, and to propose an integrative conceptual framework describing their interaction. **Materials and Methods:** A comprehensive literature review was conducted using the PubMed, Web of Science, and Scopus databases. Search terms targeted the etiology and pathogenesis of adolescent idiopathic scoliosis, hemispheric lateralization, stress responses, body schema disturbances, and associated mental disorders. The review was reported in accordance with PRISMA (Preferred Reporting Items for Systematic Reviews and Meta-Analyses) recommendations. A structured qualitative synthesis of 225 relevant publications was performed. **Results:** The analyzed studies revealed several complementary conceptual approaches to AIS pathogenesis. Emerging evidence suggests that atypical hemispheric lateralization, potentially associated with right-hemisphere (RH) dysfunction, may contribute to susceptibility to AIS. Such patterns of lateralization have been linked to specific stress-related coping strategies, including harm avoidance, as well as to disturbances of body schema and an increased prevalence of certain mental disorders. Gender-related differences in stress responses and in the development and progression of AIS were consistently reported across studies. Collectively, these findings support the hypothesis that neuropsychological and stress-related mechanisms, including phenomena described as the “distorting mirror effect”, may contribute to the persistence and progression of spinal deformity in vulnerable individuals. **Conclusions:** AIS appears to be a multifactorial condition in which atypical neuroanatomical asymmetry, stress-related processes, and altered body representation interact. This integrative perspective generates hypotheses suggesting that prevention and treatment strategies for AIS could benefit from incorporating approaches aimed at modulating stress responses and enhancing brain neuroplasticity. Further interdisciplinary studies integrating clinical, neuroimaging, and neurobiological methods are warranted to clarify underlying mechanisms.

## 1. Introduction

AIS is the most common spinal deformity, developing during adolescence with a reported prevalence ranging from 0.47% to 5.2% [1]. Currently, a multifactorial model of AIS etiopathogenesis is generally accepted.

Various spinal deformities are frequently observed in disorders of the brain [2,3,4]. Evidence supporting the leading role of the central nervous system (CNS) in the development of AIS includes neuroimaging findings revealing alterations in multiple structures of the brain (the corpus callosum, cerebellum, brainstem, and cerebral cortex) and spinal cord in patients with AIS [5,6,7,8,9,10,11,12], data on somatosensory dysfunction and its impact on postural balance control [13], as well as the frequent coexistence of scoliosis with certain brain disorders.

Compelling support for this concept is provided by the study reported in [14], in which asymmetry in the electromyographic (EMG) activity of the paraspinal muscles persisted following surgical correction of spinal deformity and spinal fusion, remaining significantly different from that observed in healthy control subjects. Consistent with these findings, the authors of [15] proposed that scoliosis originates from an unrecognized CNS dysfunction, with spinal curvature representing its external manifestation rather than the primary pathology.

A correlational relationship is observed between atypical lateralization and AIS [16,17]. A pronounced leftward shift in lateralization may be associated with RH dysfunction. Abnormal patterns of lateralization are considered a form of neurodevelopmental asymmetry that may adversely affect cognitive and emotional development in children [18].

High levels of stress during adolescence creates risks for the development of mental health problems across a wide spectrum of disorders. Mental disorders occur more frequently in individuals with AIS than in the healthy population, while a higher prevalence of spinal deformities is observed among individuals with psychiatric disorders [19,20]. We hypothesize that this may indicate the presence of certain shared developmental mechanisms underlying AIS and mental disorders. Moreover, mental disorders are not necessarily only a consequence of AIS, as they are considered in most studies. Psychological disturbances may instead develop earlier and, under the influence of stress during the sensitive adolescent period, potentially contribute to the development of scoliosis.

**Figure 1:**
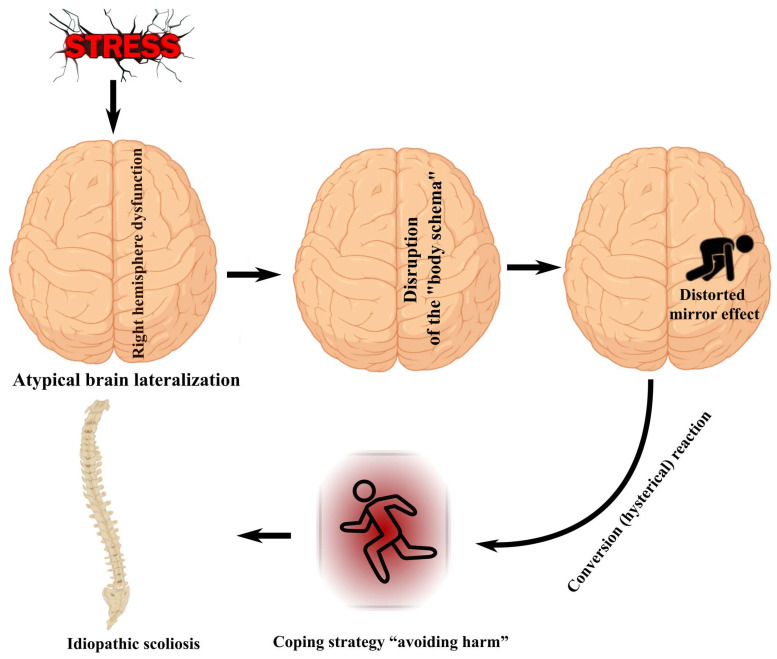
Proposed hypothesis. Stress, in combination with atypical lateralization and RH dysfunction, is accompanied by the development of a harm-avoidance strategy and scoliosis. Disturbance of the body schema and the “distorting mirror effect” contribute to the maintenance of scoliosis.

Based on the analysis of available scientific data, we propose the following hypothesis: AIS represents a stress-related conversion reaction of the spine, functioning as a harm-avoidance coping strategy in the context of atypical lateralization and RH dysfunction. Disturbances of the body schema resulting from this dysfunction, together with the emergence of the “distorting mirror effect”, may act as maintaining factors that contribute to the persistence and progression of scoliosis (Figure 1).

The “distorting mirror effect”, as used in the present work, refers to a hypothetical neurocognitive phenomenon characterized by a mismatch between actual somatic and postural states and their internal representation within the body-schema network. Unlike classical disturbances of body image, which primarily concern conscious visual or affective evaluation of one’s appearance, the proposed effect operates at the level of sensorimotor integration and implicit bodily awareness. It is hypothesized to arise from atypical hemispheric lateralization combined with relative RH dysfunction, leading to insufficient monitoring and correction of distorted bodily signals by left-hemispheric interpretative mechanisms. Importantly, this construct is conceptual and currently lacks direct empirical validation; its potential verification would require experimental paradigms combining neuroimaging, sensorimotor perturbation, and body-perception tasks.

It should be emphasized that the proposed associations between atypical lateralization, stress, neuropsychological features, and AIS are based predominantly on correlational and indirect evidence. The present model is therefore hypothesis-generating rather than confirmatory and does not imply direct causality. To test this hypothesis, we conducted a study of the relationships between AIS and neurophysiological, psychological, biomechanical, anthropometric, and stress-related factors, with subsequent conceptualization of the obtained results.

The aim of this study is to synthesize and critically integrate existing evidence from neurobiology, psychology, biomechanics, and orthopedics in order to develop a hypothetical conceptual framework for the development and progression of AIS. Specifically, the review focuses on examining the potential roles of atypical brain lateralization, stress-related neuropsychological mechanisms, disturbances of the body schema, and sex-specific factors, as well as on conceptualizing how the interaction of these processes may contribute to the emergence and persistence of three-dimensional spinal deformity in a subset of patients.

## 2. Methods

### 2.1. Study Design and Data Search

This study was conducted using a systematic literature search in accordance with the PRISMA guidelines. However, due to the interdisciplinary nature of the research question and the necessity to integrate heterogeneous sources—including experimental studies, clinical observations, theoretical models, and hypothesis-driven publications—the present work should be regarded as an integrative narrative review rather than a classical systematic review. Formal meta-analysis and quantitative risk-of-bias assessment were not feasible because of substantial heterogeneity in study designs, outcome measures, and conceptual frameworks.

The literature search was performed in the PubMed, Scopus, PsycInfo, MEDLINE, Web of Science, and Google Scholar databases, covering the period from 1965 to February 2025, with an emphasis on studies published after 2000 and additional screening of the reference lists of included articles. The search keywords included “adolescent idiopathic scoliosis”, “brain lateralization”, “stress”, “three-plane spinal deformity”, “gender differences”, “body schema”, “conversion disorder”, and related terms. Data on neurophysiological, psychological, biomechanical, and anthropometric factors associated with AIS were synthesized.

### 2.2. Inclusion and Exclusion Criteria

Reviews, experimental studies, hypotheses, case reports, and commentaries conducted in humans or animal models that addressed the association between AIS and neurophysiological, psychological, biomechanical, and anthropometric aspects were included. The selection criteria required full-text articles published in English that contained qualitative or quantitative data. Studies lacking clear data, addressing irrelevant topics, or representing duplicate publications were excluded.

### 2.3. Study Selection

**Figure 2:**
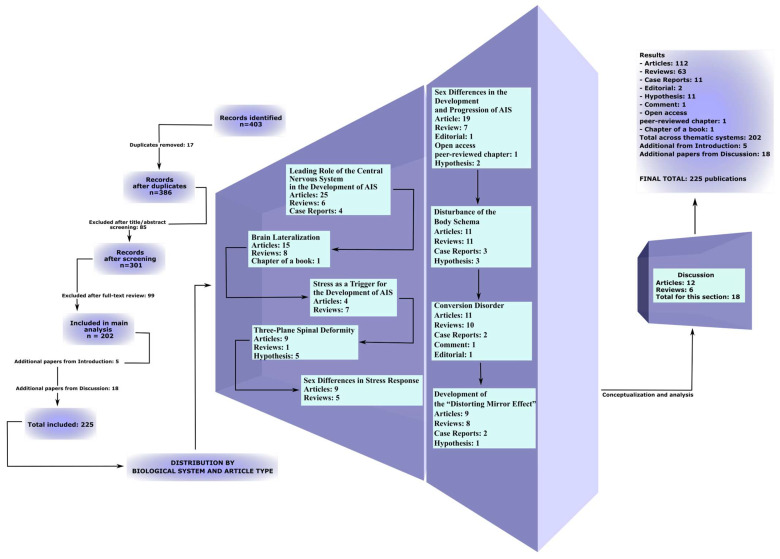
Expanded PRISMA-ScR flowchart illustrating the process of study identification, screening, eligibility assessment, and inclusion in the review, with details by publication type in the relevant sections.

The study selection process consisted of several stages. Initially, titles and abstracts were independently screened by the reviewers, followed by full-text evaluation for relevance and methodological quality. The final dataset comprised 202 studies, including 35 addressing the leading role of the CNS in the development of AIS, 24 on brain lateralization, 11 on psychological stress, 15 on three-plane deformity, 14 on sex differences in stress response, 30 on sex differences in AIS development, 28 on disturbances of body schema, 25 on conversion disorder, and 20 on the development of the “distorting mirror effect”. In addition, 5 sources were included in the Introduction and 18 in the Discussion, resulting in a total of 225 publications included in the review (Figure 2, Supplementary Materials Table S1).

The included body of literature consisted predominantly of original research articles represented across all sections of the review. In addition, review articles, case reports, editorials, hypotheses, book chapters, and peer-reviewed open-access chapters were included to complement the corresponding thematic sections of the analysis (Figure 2, Supplementary Materials Table S1).

### 2.4. Data Extraction

Data were extracted using a standardized form that included study type, number of participants, sex and age, methods, and key findings related to AIS. Disagreements were resolved by consensus.

### 2.5. Quality Assessment

Study quality was assessed using established methodological criteria appropriate to each study design. Review articles were evaluated using structured critical appraisal principles, experimental studies were assessed according to observational quality standards, and hypotheses and case reports underwent qualitative critical evaluation. Studies were categorized as high, moderate, or low quality, and this classification was taken into account during interpretation of the findings.

### 2.6. Data Analysis

Due to the heterogeneity of study types and topics, data analysis was performed as a qualitative synthesis. The results were systematized into eight main sections and presented in a coherent narrative reflecting the neurophysiological mechanisms, triggers, and biomechanical features of AIS. The narrative format allowed integration of heterogeneous data arising from differences in methodologies, samples, and availability of quantitative indicators across the included sources, thereby providing a comprehensive view of the complex interrelationships among the studied aspects and the multifactorial nature of AIS.

## 3. Results

### 3.1. Leading Role of the Central Nervous System in the Development of AIS

The leading role of the CNS in the development of AIS has been established. The principal mechanisms are considered to be abnormal interhemispheric asymmetry, impairments in sensorimotor integration and postural control, and structural as well as functional alterations in various brain regions.

Various spinal deformities are frequently observed in disorders of the brain [2,3,4,20,21,22,23,24,25,26,27,28,29,30,31]. Consistent with this observation, AIS is associated with both functional and structural brain changes [5,6,7,8,10,32,33,34,35,36]. These include asymmetrical bioelectrical activity with predominance of the left-hemisphere (LH) [32], reduced fractional anisotropy in the corpus callosum [7,34], and decreased white matter volume in interhemispheric regions [6]. These changes correlate with the severity of scoliosis (*p* < 0.05). In AIS, an increased cerebellar volume and reduced cortical thickness in motor-related regions have also been demonstrated (Table 1) [8,10,14,35].

Neuroimaging data indicate impaired sensorimotor integration in patients with AIS [13,32,35,36,37,38]. Hyperactivation of the supplementary motor area (*p* < 0.001) and reduced corticocortical inhibition on the concave side have been identified [13,36,38]. In experimental models, scoliosis was induced in 25–53% of animals by lesions of the brainstem or spinal cord (Table 1) [11,39,40,41].

A frequent comorbidity of AIS is cerebellar tonsillar descent (48%, *p* < 0.00001), while severe curvatures are often associated with syringomyelia (33.3% in severe deformities, *p* < 0.05) (Table 1) [8,9,42].

### 3.2. Brain Lateralization

The significance of brain lateralization in the development of AIS has been demonstrated. Up to the age of three years, RH dominance prevails; subsequently, the vector of lateralization shifts to the left, and during adolescence shifts again toward the right (Table 2) [43,44,45]. Structural differences in the anterior cingulate cortex (ACC) and the corpus callosum between right- and left-hemisphere–dominant individuals further confirm the importance of lateralization [46]. Depression and post-traumatic stress disorder (PTSD) are associated with RH dysfunction [47,48], whereas suicidality is linked to a compensatory shift toward LH dominance [49].

The LH predominantly mediates language functions, while the RH is responsible for visuospatial processing [50]. Individuals with AIS exhibit abnormal lateralization patterns. In adolescents with AIS, the risk of developing schizophrenia is increased by 52% (HR 1.52, 95% CI 1.03–2.23, *p* = 0.04) [20], and the prevalence of mental disorders reaches 7%, compared with 4–5% in control groups (OR 1.47–1.74, *p* < 0.001) [19]. Parents of patients with AIS demonstrate higher rates of depression and anxiety (14.1% vs. 3.5–4.7%, *p* < 0.05), which correlate with deformity severity (OR 8.26 for Cobb angle ≥ 50°, *p* = 0.034) [51]. Neuroticism and introversion are more common in patients with AIS (r = −0.51 to −0.60, *p* < 0.01) (Table 2) [52,53,62].

An ectomorphic somatotype predominates in the AIS group, whereas an endomorphic somatotype is more common in controls [54,55,56,57]. Girls with AIS show anthropometric parameters below normative values (BMI 20.1 vs. 21.4, *p* < 0.001), and 21.2% have a BMI < 17.5 (Table 2) [55], which may reflect increased stress sensitivity [63].

Atypical lateralization negatively affects a child’s mental health [18,58,59,64]. No correlation has been found between psychopathology in AIS and scoliosis severity [60]. The influence of lateralization on personality traits is further supported by differences in cognitive and behavioral styles between right- and left-hemisphere–dominant individuals across 30 categories (*p* < 0.05) (Table 2) [61].

### 3.3. Stress as a Trigger for the Development of AIS

The impact of stress during adolescence on neuronal maturation trajectories and on functional and structural brain changes associated with AIS has been confirmed. Active development of limbic and cortical brain regions contributes to heightened stress susceptibility during adolescence [65,66,67]. Early life stress (ELS) leads to reduced hippocampal volume and hyperreactivity of the amygdala (*p* < 0.05) [68,69], which is associated with cognitive and emotional impairments [70] and an increased risk of mental disorders (OR 1.5–3.0, *p* < 0.05) (Table 3) [71]. Gender differences in brain development modulate stress responses [72].

In girls exposed to ELS, reductions in hippocampal, corpus callosum, and frontal cortex volumes have been identified, with distinct vulnerability windows for each brain region [73]. Consistent with these findings, experimental studies in mice demonstrate that ELS induces lifelong hypersensitivity to stress (Table 3) [74].

Stress may adversely affect sensorimotor integration and postural control [48,68]. The absence of a strong correlation between stress type and awareness (r < 0.3, *p* < 0.05) indicates substantial individual variability (Table 3) [75].

### 3.4. Three-Plane Spinal Deformity

The leading role of three-plane spinal deformity in the pathogenesis of AIS has been confirmed. The spiral arrangement of muscles and axial loading may contribute to spinal instability in AIS [76,77,78,79]. Infantile idiopathic scoliosis (IIS) and AIS differ in their clinical presentation, underscoring age-specific characteristics of the condition [80].

Hand dominance, as well as the position and gravitational influence of the heart and aorta, determine the lateralization of the convex spinal curve [81]. Age and sex affect posture, with a predominance of kyphosis in boys and lordosis in girls (*p* < 0.05) [82]. A congenital pattern of vertebral rotation changes direction with age and corresponds to the direction of scoliotic curves (*p* ≤ 0.001) [83,84]. Vertebral rotation is also influenced by body position (Table 4) [85].

The direction of the scoliotic curve correlates with the anatomy of internal organs (*p* < 0.001) [86]. In dextrocardia, left-sided thoracic scoliosis is observed (*p* = 0.00003) [87]. The center of mass of the thoracic cage shifts from the right in infancy to the left during adolescence (r = 0.77, *p* < 0.001) (Table 4) [88].

Three-plane deformation represents a universal compensatory mechanism of the spine [89]. Gender differences in vertebral rotation are pronounced in infancy (*p* = 0.023) but are no longer evident during adolescence [83]. In children with mild idiopathic scoliosis (Cobb angle < 25°), changes in curvature patterns (*p* > 0.05) are likely explained by mechanisms of spinal tuning and balancing (Table 4) [90].

### 3.5. Sex Differences in Stress Response

Physiological, psychological, and neurobiological sex differences that influence susceptibility to stress-induced conditions, including postural changes, have been identified.

Women exhibit lower activity of the hypothalamic–pituitary–adrenal (HPA) axis and the autonomic nervous system (ANS) (*p* < 0.05), along with more pronounced negative affective responses [91,92]. These differences are associated with estrogen, which attenuates sympathoadrenal reactivity [93]. In men, risk-taking behavior (d > 0.20 for 14 of 16 behavioral domains) is enhanced under stress, whereas women tend to avoid risk, which may represent an evolutionary strategy (Table 5) [94,95]. Avoidance of active stress resolution is maladaptive and predisposes individuals to psychosomatic disorders [96]. Women demonstrate increased stress sensitivity, which is associated with a higher risk of affective disorders [97]. These differences may contribute to stress-induced postural alterations.

Stress increases LH activity (*p* < 0.05), suppressing negative emotions [98]. Women more frequently exhibit sadness and anxiety following stress exposure (*p* < 0.05) [92], perceive life events as less controllable and more negative (*p* < 0.05), and more often employ emotional and avoidant coping strategies, which are associated with psychological distress and somatic symptoms [99]. The morphology of the right anterior cingulate gyrus (ACG) correlates with fear proneness, anxiety, and harm-avoidance coping strategies in women [100,101]. Emotional coping is associated with PTSD (*p* = 0.004) and dissociation (*p* < 0.04) [102], whereas avoidance-related coping is linked to reduced connectivity of brain networks (*p* < 0.05) [103]. In men, an optimistic personality profile is associated with greater integrity of RH white-matter tracts (*p* < 0.05), indicating higher stress resilience (Table 5) [104].

### 3.6. Sex Differences in the Development and Progression of AIS

Anatomical, hormonal, biomechanical, and neurophysiological factors underlying sexual dimorphism in AIS have been identified, with a higher prevalence and greater progression observed in girls.

Adolescence is accompanied by an increase in rotational spinal instability [105,106,107]. A smaller vertebral cross-sectional area (CSA) in girls (7.93 ± 0.69 vs. 9.38 ± 1.46 in boys, *p* < 0.0001) and a more pronounced lumbar lordosis (27.6 ± 8.0° vs. 23.7 ± 6.1°, *p* = 0.02) increase spinal flexibility and reduce stability, thereby elevating the risk of deformity development [106,107,108,109,110]. Estrogen, by decreasing connective tissue stiffness, contributes to the progression of AIS [111,112,113,114,115,116,117]. Joint hypermobility (JH), which is more common in girls (*p* < 0.05), is associated with AIS and anxiety [118,119,120,121,122]. Girls reach the peak spinal growth earlier, increasing their vulnerability to deformity progression [123,124]. An ectomorphic somatotype, typical of girls with AIS, is associated with reduced postural stability and an increased risk of progression (*p* < 0.05) [125,126,127]. Dorsal shear loads and asymmetric growth of the vertebral neurocentral cartilage are considered leading mechanisms in AIS progression [128]. In contrast, a more pronounced thoracic kyphosis in boys (*p* < 0.0001) and higher tendon collagen synthesis (*p* < 0.05) provide greater rotational stability [129,130] and contribute to overall spinal stability (Table 6) [117,131].

Sex-related differences in brain development—larger brain volume and more pronounced interhemispheric asymmetry in males, earlier peak brain volume in females (*p* < 0.05)—as well as differences in neuronal asymmetry (*p* < 0.02) and interhemispheric connectivity (*p* < 0.05), may influence motor coordination and postural stability (Table 6) [72,132,133,134].

### 3.7. Disturbance of the Body Schema

Disturbances in body representation in AIS have been shown to affect self-perception, motor control, and mental health.

The body schema is a dynamic neural construct that integrates multisensory information to regulate posture, motor behavior, and spatial orientation [135,136,137,138,139,140,141]. Spatial neglect [142] and the phantom limb phenomenon [143,144,145] are associated with body schema disturbances and highlight the role of the right parietal cortex in bodily awareness [146,147,148]. The existence of aplastic phantoms (*p* < 0.01) supports the innate nature of body representation [144,145,149,150,151]. The body schema continues to develop from early childhood [152], is vulnerable in preterm infants [153], and remains particularly sensitive during adolescence. Adolescents with AIS, compared with controls, demonstrate significant disturbances in body image (*p* < 0.005) and body schema (*p* < 0.05) (Table 7) [154,155,156,157,158].

A hypothesis has been proposed that delayed maturation of the body schema contributes to AIS progression due to the inability of the CNS to adequately control asynchronous skeletal growth [161].

Concepts such as “proprioceptive memory” have been proposed to explain phantom bodily sensations [162], alongside evidence supporting the dominant role of the RH in bodily self-awareness [148]. Lesions of the RH are associated with spatial neglect and egocentric deviation [159], whereas LH lesions are linked to disturbances of the body schema (27.2%, *p* < 0.05) (Table 7) [160].

### 3.8. Conversion Disorder

The role of stress, neurobiological mechanisms, and sex differences in the development of conversion disorder (CD), as well as its possible association with psychosomatic aspects of AIS, has been established.

CD is characterized by functional neurological symptoms occurring in the absence of organic pathology [163] and has a relatively high prevalence [164,165]. Stress and abuse (OR 5.6, 95% CI 2.4–13.1) are the main risk factors for CD, although in some cases identifiable stressors are absent [166,167,168]. Notably, CD affects women significantly more frequently than men (Table 8) [169,170,171,172,173].

Neuroimaging studies have demonstrated the involvement of the ACC, amygdala, and basal ganglia in stress-related mechanisms [174,175,176,181,182,183]. In CD, left-sided symptom localization is more frequently reported, although findings are inconsistent (*p* > 0.05) [177,184], and right-hemisphere abnormalities are observed in up to 71% of cases (*p* < 0.02) (Table 8) [178]. Structural alterations in cingulo-insular regions suggest stress-induced neuroplasticity [185,186].

The diversity of CD symptoms is presumed to reflect the functional heterogeneity of the ACC [187]. Self-injurious behavior is interpreted as a maladaptive strategy for reducing uncertainty through “acting on the body,” which overlaps conceptually with the psychosomatic aspects of AIS. RH dysfunction is considered a key factor underlying self-injury [49]. Self-injurious behavior is associated with low oxytocin levels and high pain tolerance (OR 0.55–1.67, *p* < 0.021) (Table 8) [179,180].

### 3.9. Development of the “Distorting Mirror Effect”

Neural, cognitive, and biomechanical characteristics of the “distorting mirror effect”—defined as a distorted perception of the body and peripersonal space—and its potential association with AIS have been identified.

Alterations in the balance of complementary hemispheric functions during reasoning lead to distorted perception [188,189,190,191]. The RH, particularly the ventromedial prefrontal cortex (vmPFC), increases uncertainty in decision-making, with more pronounced impairments in women following left-sided lesions (*p* < 0.05) [192]. The dorsomedial prefrontal cortex/dorsal anterior cingulate cortex (dmPFC/dACC) is involved in body perception and conflict processing, mechanisms that may overlap with those implicated in AIS [193,194]. RH dysfunction predominates in delusional syndromes (*p* < 0.05), in which hyperactivity of the LH generates false explanations (Table 9) [195,196,197,198].

Caloric vestibular stimulation, by modulating activity in the temporoparietal, insular cortices, and the ACC, transiently attenuates perceptual distortions (Table 9) [199,207].

Horizontal eye movements enhance the perception of trunk rotation [200], while cognitive conflict leads to activation of the dACC (*p* < 0.0001) [194,201]. Lesions of the ACC impair the integration of reward and risk [202]. Under conditions of unilateral hemispheric suppression following electroconvulsive therapy, the RH preserves contextual perception, whereas the LH operates on abstract inferences [203,204]. In rats, cerebellar asymmetry induces postural asymmetry [205], and asymmetric expression of the estrogen receptor gene Esr1 in individuals with AIS exacerbates scoliosis severity (*p* < 0.05) (Table 9) [206].

## 4. Discussion

The present review represents an attempt to elucidate the complex nature of AIS by identifying the contribution of neurophysiological, psychological, and biomechanical factors to the development and progression of this condition. Importantly, this work is hypothesis-generating in nature and aims to integrate converging but largely indirect lines of evidence rather than to establish definitive causal relationships. The proposed framework differs conceptually from earlier “vicious cycle” models of idiopathic scoliosis, which primarily focused on peripheral biomechanical feedback mechanisms without incorporating central neurocognitive processes. In contrast, the present hypothesis emphasizes the role of brain lateralization, stress-related neuroplasticity, and body-schema representation as modulatory factors rather than primary structural causes. Thus, the present model should not be interpreted as a revival of earlier functional loop theories, but rather as an expanded integrative framework incorporating contemporary findings from neuroscience and stress research. The model is therefore intended as an integrative and exploratory framework, not as a replacement for established biomechanical or neurodevelopmental theories. Accordingly, it is proposed as a conceptual tool to guide future experimental and clinical research rather than as a definitive explanatory theory of AIS.

Table 10 summarizes the proposed integrative, multilevel model of AIS, illustrating how processes operating across genetic–hormonal, tissue, biomechanical, sensorimotor, cortical, interhemispheric, cognitive–perceptual, emotional, and behavioral levels may converge to influence the development and persistence of spinal deformity. Importantly, the model does not imply a linear or strictly causal sequence of events; rather, it conceptualizes AIS as the outcome of interacting vulnerabilities and modulatory mechanisms that may become functionally coupled during critical developmental periods. At the central nervous system level, disturbances in interhemispheric balance, body-schema representation, and error-monitoring circuits are proposed to influence sensorimotor integration and postural control, thereby shaping biomechanical loading patterns of the spine. These central mechanisms interact with sex-specific hormonal, tissue, and growth-related factors, potentially amplifying asymmetry under conditions of stress and rapid growth. Within this framework, phenomena such as the “distorting mirror effect” are viewed as higher-order perceptual–cognitive manifestations of earlier functional distortions, contributing to reduced bodily awareness, delayed corrective responses, and reinforcement of maladaptive motor patterns. Collectively, the table provides a conceptual scaffold linking brain-level processes to spinal morphology, highlighting AIS as a dynamic, multilevel condition rather than a disorder driven by a single etiological factor.

Hemispheric lateralization plays a central role in body perception and postural control. The LH functions as an “interpreter” of events: it seeks to eliminate uncertainty, draws conclusions, and proposes hypotheses, often disregarding contradictions or implausibility in its own explanations. In contrast, the RH detects and resolves conflicts between proposed explanations and reality [188,189].

In the healthy brain, these complementary modes of information processing interact dynamically, supporting higher-order cognition and coordinated motor control. However, atypical patterns of hemispheric lateralization, particularly when accompanied by relative RH dysfunction, may disrupt this balance and contribute to disturbances in body perception and postural regulation. Such disturbances are not assumed to be universal in all individuals with AIS, but may characterize a specific vulnerable subgroup with particular neurodevelopmental and stress-related profiles. Experimental studies involving transient inactivation of the RH during electroconvulsive therapy have shown that the LH, unlike the RH, becomes detached from reality and generates false conclusions [204]. Functional insufficiency of right-hemispheric mechanisms underlies maladaptation and many mental and psychosomatic disorders. Within the present framework, AIS is not conceptualized as a direct consequence of these mechanisms, but as a possible somatic context in which they may manifest or exert modulatory effects.

Stress is a common feature of childhood and adolescence, developmental periods during which the brain continues to undergo active maturation [65]. ELS reduces brain stress resilience [68,69,70,71,74,75], adversely affects the RH, and promotes the development of ineffective stress-coping mechanisms [48]. Adolescence is considered a period of increased behavioral and psychiatric vulnerability due to stress-induced alterations in neuronal maturation trajectories [66,67,208]. The developmental trajectories of different brain structures vary over time, resulting in “windows of heightened stress sensitivity” [73]. Stress triggers a tension response across multiple physiological systems, including the muscular system, leading to physical changes. The transformation of psycho-emotional tension into asymmetric muscle tone represents a psychosomatic pathway linked to the biomechanical characteristics of the spine that underlie AIS. This pathway is proposed as one of several interacting mechanisms and should not be interpreted as a primary or exclusive driver of spinal deformity.

The spine is an integral participant in the organism’s response to stressors. Three-plane spinal deformity can be viewed as a manifestation of spiral organization, a phenomenon widely observed in living systems, such as the DNA helix. In humans, symmetrically arranged groups of muscular spirals in the limbs and trunk exert opposing actions, with right- and left-handed rotational components [76,77,78]. Alterations in the balance of these muscular spirals may lead to torsion of the trunk to the right or left. Trunk and spinal rotation constitute natural movements as part of a defensive response to physical threat. Depending on the selected behavioral strategy, the initial response involves trunk extension or flexion, followed by rotation and lateral bending. Evidence for the existence of an innate spinal rotation pattern may be provided by the rotation of the thoracic vertebrae in the normal spine: to the left in childhood and to the right during adolescence. This rotational template determines the shift in the predominant direction of thoracic scoliotic curves in idiopathic scoliosis—from left-sided in infancy to right-sided in adolescence [79,83,84,85]. These observations suggest the presence of developmentally regulated biomechanical predispositions that may interact with central regulatory mechanisms.

Features of a shift in the laterality vector may contribute to a small proportion of the development of severe forms of IIS and AIS. In this context, lateralization-related mechanisms are considered modulatory rather than determinative, potentially influencing susceptibility, progression, or compensatory capacity rather than acting as primary etiological factors. With well-developed right-hemispheric mechanisms, scoliosis may fail to develop or progress. In infantile idiopathic scoliosis, the age-related reversal of the intrinsic spinal rotation pattern during growth may play a protective role. Study [90] points to the presence of compensatory mechanisms of the organism in mild idiopathic scoliosis, leading to its regression or stabilization. This supports the notion that central and peripheral adaptive processes may mitigate deformity development in a substantial proportion of cases.

Existing sex differences in stress responses [91,92,93,209] and in scoliosis development represent another link in the pathogenesis of AIS. Differences in brain developmental trajectories and activation levels between females and males [72] may underlie differential risks of mental disorders.

In terms of growth and development during childhood and adolescence, the female brain matures 1–2 years earlier than the male brain [72,123,134]. However, rapid maturation may prevent neural structures from achieving a high level of functional refinement, rendering them more vulnerable. Boys exhibit earlier development of RH mechanisms, whereas girls demonstrate earlier development of LH functions [132]. Accordingly, boys are more right-hemisphere–dominant in childhood. The authors of [133] reported greater interhemispheric connectivity in girls and greater intrahemispheric connectivity in boys. This organization is thought to facilitate efficient coupling of perception and coordinated action in males, while in females it may promote enhanced interaction among analytical brain centers. Such differences may contribute to sex-specific patterns of stress processing and postural regulation.

Stress elicits activation of different brain structures and engagement of distinct coping strategies in males and females. Boys tend toward a fight-oriented response, making decisions aimed at positively changing the situation and mobilizing all available resources to achieve this goal. Girls are more inclined toward passivity and compromise; under stress, they tend to withdraw from the problem either physically or psychologically. This approach contributes to less constructive coping strategies. Boys demonstrate higher levels of exploratory activity, whereas girls exhibit predominantly stereotyped activity patterns. Under stress, exploratory behavior in boys increases, with a preference for non-standard actions, which are considered the most adaptive. In contrast, girls tend toward stereotyped behaviors both under stress and in calm conditions [96].

A study [100] found that in women, the volume of the right ACG relative to the left was significantly greater than in men. The authors concluded that the right ACG represents the anatomical substrate of the typically “female” harm-avoidance coping strategy. Avoidance-oriented coping correlates with introversion, is associated with emotional distress, and predisposes individuals to the development of mental disorders such as PTSD, anxiety, major depression, and suicidal behavior [102,103,210]. These characteristics of the female stress-response pattern render women more vulnerable to psychosomatic postural changes.

Progression of AIS occurs during the growth spurt, a period when increased demands are placed on the organism, including the spine, which it does not always successfully meet [105]. This period is also associated with a higher risk of mental disorders and with sex differences in their prevalence [211,212]. An additional risk factor may be a genetic predisposition to heightened stress reactivity [213]. The convergence of rapid growth, stress exposure, and neurodevelopmental vulnerability may therefore represent a critical window for AIS progression.

Compared with boys, the spine in girls is taller, thinner, and more flexible [124]. Consequently, it bends more readily in the frontal plane than in the sagittal plane, where stiffness is relatively preserved [214]. Intervertebral disks are characterized by greater height [106], and thoracic kyphosis is less pronounced [110,129]. Flattening of thoracic kyphosis is accompanied by increased rotational spinal instability, facilitating scoliotic curvature in the frontal plane. The mid- and lower thoracic segments are more posteriorly displaced, thereby increasing the likelihood of vertebral rotation, particularly during the period of maximal growth velocity in puberty [85,128,130,215,216,217]. In girls with an ectomorphic somatotype, the tendency toward posterior deviation is more pronounced than in controls [125,126]. Girls experience an earlier growth spurt, with peak growth velocity coinciding with the minimal thoracic kyphosis angle, whereas in boys it coincides with maximal kyphosis. As a result, adolescent scoliosis develops against a background of predominantly lordotic posture in girls and kyphotic posture in boys. Increased kyphosis is considered more favorable, as the degree of scoliosis is then substantially lower.

The elasticity and plasticity of tissues differ qualitatively and quantitatively between women and men. A possible explanation lies in the differential effects of sex hormones on these tissues, specifically increased stiffness under the influence of testosterone, whereas estrogen exerts the opposite effect. Women exhibit lower mechanical strength of tendons, reduced rates of collagen synthesis in tendons following physical loading, and an absence of tendon hypertrophy after training [111,112,121].

JH is frequently observed in AIS, particularly in girls [118,122]. Adolescents with AIS are typically characterized by an ectomorphic somatotype and personality traits such as introversion [52,54,55,57,62]. JH is associated with ectomorphic somatotype, anxiety, depression, eating disorders, neurodevelopmental disorders, and stress-sensitive psychosomatic conditions [119,127,218,219].

**Figure 3:**
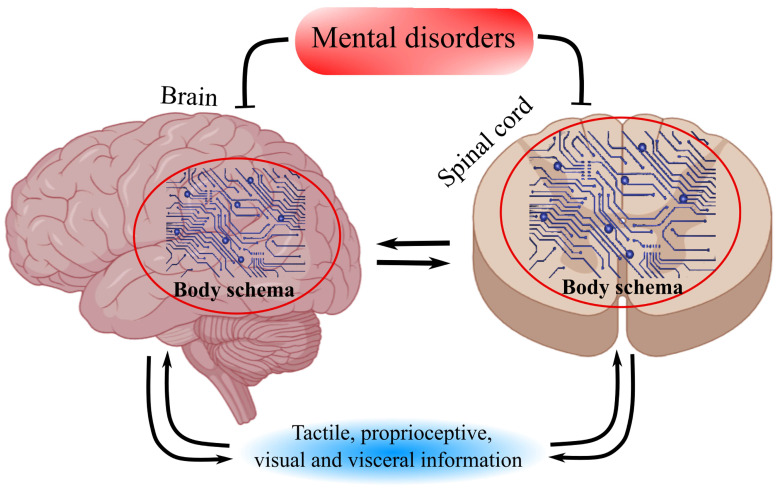
Disruption of the Body Schema. The “body schema” is localized in the brain and spinal cord, with some components being innate. It is updated based on information from various sources and is disrupted in mental disorders.

Adolescents with AIS exhibit poorer body awareness compared with their healthy peers [37,154,155,156,157,158], which may be related to RH dysfunction, given its role in integrating proprioceptive information [146,147] (Figure 3). Disturbances of the body schema in AIS are associated with psychological distress [155].

Cases of phantom limb phenomena in individuals with congenital limb absence or early-life amputation, as well as the ability of newborns to imitate facial gestures, indicate the innate nature of the body schema [143,144,145,149,150,152]. Throughout life, the body schema is continuously updated based on incoming tactile, proprioceptive, visual, and interoceptive information [140]. At the same time, it is vulnerable to various influences [140,153]. Disturbances of the body schema are observed in a range of mental disorders [220]. We propose that atypical lateralization with subsequent RH dysfunction and disruption of the body schema represents a factor sustaining AIS. This sustaining role is conceptualized as functional and dynamic rather than structural, interacting with ongoing growth-related biomechanical processes.

The link between perception and physical manifestations finds a logical extension in CD. Childhood stress correlates with the development of CD [166,167,171,172]. The possibility of spinal deformity following psychological stress is illustrated by cases of psychogenic camptocormia [22]. In women, CD, as well as mild psychiatric morbidity, occurs more frequently than in men [170,173]. Conversion symptoms are more frequently observed on the left side of the trunk, which suggests a possible dysfunction of the RH of the brain [177,182,184,221]. RH dysfunction resulting from atypical lateralization may contribute both to the development of conversion disorders and to disturbances of the body schema [178,181]. Neuroimaging studies of CD frequently reveal abnormalities in the cingulate gyrus [174,176,183].

Stress, by activating the ACC, may suppress motor functions, transforming emotional distress into physical symptoms. In women, this relationship is further amplified by experiences of sexual abuse, pointing to a shared pathway between CD and AIS, in which neuroplasticity of the cingulo-insular regions plays a central role [185].

Dysfunction of the ACC has been linked to a range of mental disorders, including schizophrenia, obsessive–compulsive disorder, depression, bipolar disorder, PTSD, and autism spectrum disorders [186,187,222,223].

As noted above, the right ACG is responsible for the harm-avoidance coping strategy. Thus, a possible link can be traced between harm-avoidance coping under stress in individuals with atypical lateralization and the emotional–motor component required for its implementation. In this context, scoliosis may be regarded as an orthopedic manifestation of a conversion reaction of the organism. This interpretation is not intended to reclassify AIS as a psychiatric disorder, but rather to highlight potential overlaps in stress-related neurobiological mechanisms shared with other functional somatic conditions.

AIS may be compared to paradoxical behavior such as non-suicidal self-injury, which is likewise maladaptive and expressed through inappropriate means. Such behavior is thought to arise from functional insufficiency of the RH [49]. Paradoxical behavior is common in adolescence, and in approximately 80% of cases is accompanied by various mental disorders. AIS, as a specific variant of paradoxical behavior, by “acting on the body,” reduces the degree of uncertainty, including within internal models of the body and personality. Among the positive effects of maladaptive behavior are relief of emotional stress and negative affect [179,180,224,225]

**Figure 4:**
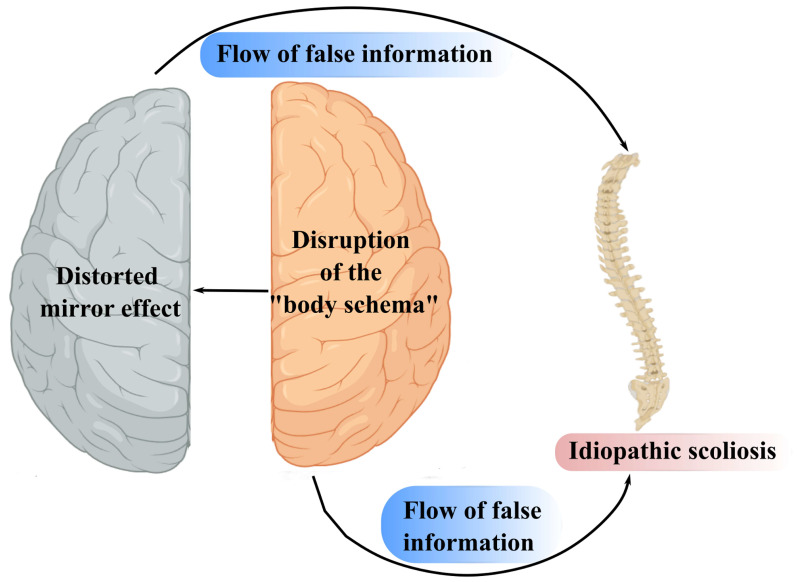
Development of the Distorted Mirror Effect. When the “body schema” is disrupted, false information from the right hemisphere is transmitted to the left hemisphere and accepted as accurate due to the distinct reasoning strategies of each hemisphere.

The proposed psychosomatic mechanism underlying the development of AIS leads us to the concept of the “distorting mirror effect”, illustrating how abnormal lateralization results in distorted perception and integrates the neural and biomechanical aspects of AIS into a unified framework (Figure 4).

Under conditions of RH dysfunction, erroneous information about the body schema is accepted without adequate monitoring or correction. To some extent, this resembles delusional belief formation [191,195,197,198]. Notably, RH dysfunction has been reported both in delusional states and in CD, and stimulation of the RH—for example, via left-sided caloric vestibular stimulation—leads to resolution of pathological symptoms in both conditions [199,207].

Based on the above considerations, we suggest a hypothesis that scoliosis could potentially be interpreted as a stress-related conversion-like response of the spine in the context of atypical lateralization and possible RH dysfunction. The organism constructs motor programs based on distorted input from the body-schema neural network, thereby maintaining the scoliotic deformity. Anatomical, hormonal, and psychological characteristics of the female organism account for their greater vulnerability. Under RH dysfunction, the adaptive capacities of the organism are reduced and insufficient to effectively resolve disturbances of postural balance.

The proposed hypothesis does not exclude the multifactorial nature of the development of AIS. Rather, it may serve as a missing link that integrates various etiological factors of AIS and the uniform three-dimensional spinal deformity through a stress-induced conversion reaction of the spine, resembling a harm-avoidance coping strategy as a possible initiator of primary scoliosis in the context of atypical lateralization and RH dysfunction.

The model of development and progression of AIS presented in this study is hypothetical and is subject to certain limitations. The contradictory nature of a number of clinical studies on AIS necessitates the development of an experimental model to confirm the role of stress as a trigger factor in the development of primary scoliotic spinal deformity, since clinical studies in humans are not feasible for ethical reasons. Computer modeling of the development of three-dimensional spinal deformity under stress conditions, particularly in childhood and adolescence, appears to be a promising approach. The incorporation of methods aimed at modulating stress-related mechanisms and activating mechanisms of brain neuroplasticity into preventive and therapeutic strategies for scoliosis treatment requires interdisciplinary research combining clinical, neuroimaging, and neurobiological approaches. Targeted experimental paradigms combining neuroimaging, sensorimotor perturbation, and longitudinal biomechanical assessment will be required to test the proposed framework.

## 5. Conclusions

1. Available evidence suggests that atypical brain lateralization, potentially associated with altered right-hemisphere functioning, may contribute to disturbances of body schema and to neurodevelopmental features observed in adolescent idiopathic scoliosis.
2. The reviewed data support a hypothesis that stress-related neuropsychological mechanisms, in the context of atypical lateralization and altered body representation, may be involved in the development and persistence of primary spinal deformity, including phenomena described as the “distorting mirror effect”.
3. The three-dimensional nature of spinal deformity in scoliosis may also be conceptualized within broader biological frameworks of rotational and spiral organization, which are characteristic of many living systems and may influence morphogenetic processes.
4. Sex-related differences in the development and progression of adolescent idiopathic scoliosis are likely influenced by sex-specific patterns of brain and spinal growth, as well as by differences in stress responsiveness and neuroendocrine regulation.
5. From an integrative perspective, preventive and therapeutic strategies for scoliosis may benefit from approaches aimed at modulating stress-related mechanisms and promoting brain neuroplasticity, warranting further interdisciplinary investigation.

## Figures and Tables

**Table 1:** Central nervous system dysfunction as a leading factor in the development of adolescent idiopathic scoliosis (AIS): clinical, neuroimaging, and experimental evidence.

Study Design/Model	Sample and Methods	Study Aim	Key Findings	Interpretation/Significance	Reference
Clinical comparative study	24 patients with Parkinson’s disease (PD): 12 with Pisa syndrome, 12 without; neuropsychological assessment (16 tests across 6 cognitive domains); assessment of trunk position perception	To compare cognitive functions in PD patients with and without Pisa syndrome	Patients with Pisa syndrome showed impairments in visuospatial abilities, attention, and language; distorted trunk position perception was observed in all patients	Suggests involvement of shared cortico–subcortical networks underlying both cognitive and postural control	[2]
Comparative MRI study	20 adolescents with idiopathic scoliosis (IS) and 26 controls; 3D-MPRAGE; volumetric analysis of 99 brain regions	To identify regional brain volume differences in IS	Significant volumetric differences in 22 regions (frontal lobes, corpus callosum, subcortical structures)	Supports the neurodevelopmental hypothesis of IS	[5]
Prospective MRI study	9 girls with left-sided thoracic IS and 20 with right-sided thoracic IS; VBM, DBM, TBM	To investigate brain asymmetries in different IS curve types	Reduced white matter volume in the genu of the corpus callosum and left internal capsule in left-sided IS	Lateralized CNS changes are associated with curve direction	[6]
Diffusion tensor imaging study	69 female patients with IS and 40 controls; ROI analysis of the corpus callosum; tractography	To assess corpus callosum microstructure in IS	Decreased fractional anisotropy (FA) in the genu and splenium; pseudo-rightward lateralization	Impaired interhemispheric integration in IS	[7]
Morphological and phase-contrast MRI study	69 female patients with IS and 36 controls	To examine cerebellar tonsil morphology, foramen magnum size, and CSF dynamics	Lower position of cerebellar tonsils and enlarged foramen magnum	Craniovertebral junction abnormalities may contribute to IS pathogenesis	[8]
Supine and upright MRI study	25 patients with IS and 18 controls	To assess the effect of verticalization on cerebellar tonsil position	Pronounced tonsillar descent in standing position in IS patients	Highlights the role of gravity and postural control	[9]
Comparative morphometric study	50 female patients with IS and 40 controls; cortical thickness analysis	To study cortical maturation in IS	Absence of normal age-related cortical thinning in IS	Impaired cortical neurodevelopment in IS	[10]
Animal model (rabbits)	Brainstem injury; electromyography (EMG)	To induce scoliosis via proprioceptive disruption	Development of scoliosis following loss of sensory afferentation	Confirms the role of proprioceptive dysfunction	[11]
Neurophysiological study	16 patients with IS and controls; transcranial magnetic stimulation (TMS)	To investigate central inhibitory mechanisms	Prolonged cortical silent period	Imbalance of CNS inhibitory mechanisms in IS	[13]
EMG study	19 patients with IS before and after spinal fusion and 15 controls	To assess paraspinal muscle function	EMG asymmetry preoperatively with partial normalization postoperatively	Muscle changes are secondary to spinal deformity	[14]
Retrospective cohort study	3702 adolescents with IS and 370,200 controls	To assess the association between IS and schizophrenia	Increased risk of schizophrenia in IS patients	Possible shared neurobiological mechanisms	[20]
Clinical observational study	300 patients with cervical dystonia	To investigate associated movement disorders	Scoliosis identified in 39% of patients	Overlapping pathogenesis of dystonia and postural deformities	[21]
Clinical case series	Adolescents and adults with camptocormia	To describe psychogenic forms	Absence of organic pathology; improvement with psychotherapy	Camptocormia may represent a manifestation of conversion disorder	[22,23,24,25]
Clinical cohort study	399 patients with anterocollis	To investigate associations of anterocollis	Association with PD, neuroleptic use, and heredity	Supports a neurogenic origin of anterocollis	[27]
Prospective study	97 patients with PD	To examine the relationship between PD and scoliosis	Scoliosis more common in women; no association with PD lateralization	Scoliosis in PD represents an independent phenomenon	[31]
EEG analysis (3DLocEEG)	Adolescents with IS	To study bioelectrical asymmetry	Shift in activity toward the left hemisphere	Accelerated development of interhemispheric asymmetry	[32]
Comparative morphometric study	12 female patients with IS and 12 controls	To compare corpus callosum analysis methods	Splenium alterations confirmed	Reliability of a multimodal approach	[33]
DTI study	10 female patients with IS and 49 controls	To assess FA of the corpus callosum	Reduced FA in the body of the corpus callosum	Impaired motor integration	[34]
Cerebellar MRI study	50 patients with IS and 40 controls	To investigate the relationship between IS and cerebellar volume and morphology	Increased volume of specific cerebellar lobules	Compensatory cerebellar remodeling	[35]
Functional MRI study	10 patients with IS and 10 controls	To examine motor activation patterns	Increased supplementary motor area (SMA) activation and asymmetry	Impaired secondary motor control	[36]
EEG and posturography	14 adolescent girls with IS and controls	To study postural control and body schema	Altered theta and alpha activity	Sensorimotor disintegration precedes deformity	[37]
Paired-pulse TMS study	9 adolescents with IS, 5 with congenital scoliosis (CS) and 8 controls	To assess corticocortical inhibition	Asymmetric reduction in inhibition	Supports the dystonic hypothesis of IS	[38]
Animal model (rats)	Brainstem nuclei lesions in 75 animals; 15 controls	To examine the link between postural control and scoliosis	Kyphoscoliosis developed in 25% of animals	Brainstem structures are critical for posture	[39]
Genetic model (mice)	20 Runx3 knockout (KO) mice and 20 wild-type (WT) littermate controls	To investigate the role of proprioception	Scoliosis in 95% of mutant mice	Proprioception is a key regulator of body axis alignment	[40]
Animal model (rabbits)	Spinal cord injury in 6 animals and costotransversectomy in 4 animals	To assess the role of muscles in IS	Myopathy secondary to deformity	Confirms secondary nature of muscular changes	[41]
Comparative MRI study	117 patients with IS and 53 controls	To assess cerebellar tonsil position	Lower tonsil position in severe IS	Severity gradient links CNS anomalies and deformity	[42]

**Table 2:** Neurodevelopment and functional hemispheric lateralization of the brain: evidence from typical development and neurodevelopmental disorders.

Study Design/Model	Sample and Methods	Study Aim	Key Findings	Interpretation/Significance	Reference
Functional near-infrared spectroscopy (fNIRS)	20 children with autism spectrum disorder (ASD; mean age 5.8 years) and 20 typically developing controls; assessment of hemodynamic responses to linguistic and non-linguistic auditory stimuli	To identify the speech-processing component underlying atypical lateralization in autism	Both groups exhibited left-hemispheric lateralization during natural speech perception; children with ASD lacked graded modulation of lateralization with decreasing linguistic relevance and showed right-hemispheric hyper-responsiveness to degraded speech	Indicates impaired hierarchical linguistic processing and atypical neural specialization	[18]
Retrospective population-based analysis	Korean National Health Insurance Database (2012–2016); ~1 million individuals per year; ~7400 children with idiopathic scoliosis (IS) annually	To assess the prevalence of psychiatric disorders in children with IS	Children with IS consistently showed a higher likelihood of psychiatric disorders compared with controls (OR 1.47–1.74; *p* < 0.001)	Suggests a systemic association between IS and psychoneurological vulnerability	[19]
Resting-state functional SPECT	Young children	To test the hypothesis of differential hemispheric maturation rates	Right-hemispheric dominance of cerebral blood flow at ages 1–3 years, followed by a leftward shift after age 3	Confirms asynchronous hemispheric neurodevelopment as a normal process	[43]
Functional MRI (fMRI)	62 right-handed men; analysis of corticocortical interactions	To investigate functional lateralization of interhemispheric interactions	The left hemisphere demonstrated greater intrahemispheric integration, whereas the right hemisphere showed more bilateral connectivity; degree of lateralization correlated with cognitive performance	Hemispheric lateralization is associated with functional efficiency of the CNS	[50]
Clinical psychometric study	64 patients with IS and their parents; 85 control families; PHQ-9, GAD-7	To assess mental health in parents of patients with IS	Parental anxiety and depression correlated with corresponding symptoms in patients; increased risk of probable major depressive disorder (pMDD) and generalized anxiety disorder (pGAD) in parents of IS patients	Highlights the familial and neuropsychological context of the disease	[51]
Cross-sectional clinical study	43 adolescents with IS; SRS-22, TAPS, 16PF-APQ	To examine personality traits and health-related quality of life in IS	Low extraversion and self-reliance; independence negatively correlated with self-image and mental health	Psychological profile may reflect CNS characteristics rather than solely a reaction to deformity	[52]
Structural MRI combined with personality questionnaires	28 healthy adults; cortical thickness and amygdala volume analysis	To investigate neuroanatomical correlates of extraversion and neuroticism	Prefrontal cortical thickness correlated with personality traits, whereas amygdala volume did not	Supports an association between personality traits and cortical architecture	[53]
Retrospective clinical study	38 patients with IS and 27 controls; somatotype assessment	To compare somatotypes in IS	Predominance of ectomorphy; negative correlation between endomorphy and Cobb angle	Morphotype may reflect neuroendocrine and neurodevelopmental characteristics	[54]
Anthropometric comparative study	52 girls with IS and 92 controls	To assess body composition and somatotype	Lower BMI, increased ectomorphy, and a higher prevalence of low BMI	Supports the hypothesis of a systemic neurobiological phenotype	[55]
Retrospective somatotype study	77 girls with IS (surgically treated cases) and historical controls	To examine the association between somatotype and IS	Significantly reduced mesomorphy in IS	Morphological profile is associated with disease presence	[56]
Prospective controlled study	52 girls with progressive IS and 62 controls	To compare morphological traits	Significantly lower mesomorphy in progressive IS	Somatotype is associated with deformity progression	[57]
Large-scale fMRI cohort (ABIDE)	964 participants (447 with ASD, 517 controls)	To assess diffuse hemispheric lateralization in autism	Reduced leftward lateralization in language and default mode networks; correlation with autism severity	Demonstrates a systemic rather than focal lateralization atypicality	[58]
fMRI study in specific language impairment	21 children with typical specific language impairment (T-SLI) and 18 controls	To investigate lateralization of language networks	Absence of left-hemispheric lateralization with right-hemispheric hyperactivation	Impaired lateralization as a neurodevelopmental phenomenon	[59]
Clinical psychopathological study	105 young men with IS and 108 controls; Korean Military Personality Inventory (KMPI)	To assess psychopathological profiles	Elevated anxiety, depressive, and psychotic scale scores in IS	Psychopathology may represent part of a systemic CNS phenotype	[60]
Behavioral study combined with MRI	150 healthy participants	To relate hemispheric dominance to behavior	Stable differences in behavioral preferences were identified	Functional CNS asymmetry has behavioral manifestations	[61]

**Table 3:** Stress as a triggering factor in the development of adolescent idiopathic scoliosis (AIS): neurodevelopmental and neurobiological evidence.

Study Design/Model	Sample and Methods	Study Aim	Key Findings	Interpretation/Significance	Reference
Cross-sectional clinical–neuroimaging study	64 unmedicated patients with major depressive disorder (MDD) and 65 healthy controls; assessment of early life stress (ELS), cognitive testing, 3T MRI	To evaluate the association between ELS, depression, cognitive function, and brain structure	Emotional and sexual abuse, as well as severe family conflict, predicted MDD; ELS was associated with cognitive impairment and reduced volumes of the orbitofrontal cortex (OFC), caudate nucleus, hippocampus, and reduced insular cortical thickness	Early stress induces persistent structural and cognitive brain alterations that increase vulnerability to psychiatric and somatic disorders	[71]
Structural MRI (sensitive periods analysis)	26 women with a history of childhood sexual abuse and 17 controls (aged 18–22 years)	To test the hypothesis of stress-sensitive developmental windows in the brain	Reduced volumes of the hippocampus, corpus callosum, and frontal cortex depending on the age at trauma exposure	Distinct brain regions exhibit specific windows of vulnerability to stress	[73]
Animal model (mice)	Maternal deprivation model; early and late postnatal stress exposure	To investigate mechanisms of long-term stress vulnerability	Early stress induced persistent transcriptional programming of the ventral tegmental area (VTA) via Otx2, resulting in increased stress sensitivity in adulthood	Stress during critical developmental periods can durably “reprogram” the CNS	[74]
Cross-sectional correlational study	929 adults; assessment of childhood trauma (CTQ) and mindfulness (FFMQ-BR)	To examine the relationship between early stress and emotional regulation	Specific types of childhood trauma were associated with alterations in particular dimensions of mindfulness, despite preserved global emotional regulation	Early stress modifies internal experience processing strategies without obligatory clinical decompensation	[75]

**Table 4:** Three-dimensional organization of the spine and congenital deformation patterns: clinical, imaging, and biomechanical evidence.

Study Design/Model	Sample and Methods	Study Aim	Key Findings	Interpretation/Significance	Reference
Population-based clinical and instrumental study	>33,000 children and adolescents (5–17 years); computer optical topography (COMOT/SOMOT); sagittal parameter analysis	To investigate age- and sex-related development of sagittal posture	Age- and sex-dependent changes in kyphosis and lordosis; minimal sex differences at age 5 with progressive divergence by age 17	The sagittal profile develops dynamically and determines conditions for 3D spinal deformation	[82]
CT study (normal spine)	146 CT scans of children aged 0–16 years without spinal pathology	To identify age-related patterns of vertebral axial rotation in the normal spine	Age-related switch in axial rotation direction (left → right); partial sex-related differences	Indicates an innate, age-dependent rotational template	[83]
CT study (normal spine)	50 adults without scoliosis; semi-automated analysis of T2–L5 vertebral rotation	To identify pre-existing rotational patterns	Upper thoracic vertebrae rotated leftward, mid- and lower thoracic vertebrae rotated rightward	The normal spine is intrinsically asymmetric in the axial plane	[84]
In vivo MRI study	30 healthy volunteers; MRI of T2–L5 in three body positions	To assess the effect of posture on axial rotation	Vertebral rotation persisted across all positions but decreased in the quadrupedal posture	Axial rotation represents a structural rather than purely postural phenomenon	[85]
Clinical observational study	198 patients with primary ciliary dyskinesia (PCD)	To evaluate the association between visceral orientation and scoliosis convexity	Curve convexity correlated with situs inversus; curvature direction opposed visceral orientation	Internal body asymmetry influences 3D spinal geometry	[86]
Population-based case–control study	8 individuals with dextrocardia and 32 controls	To assess coronal alignment of the thoracic spine	Left-sided convexity in dextrocardia versus right-sided convexity in controls	Pulsatile and volumetric asymmetries contribute to curve direction	[87]
CT study (normal spine)	155 CT scans of children aged 0–18 years; thoracic center-of-mass calculation	To investigate age-related shifts in thoracic center of mass	Progressive right-to-left shift in center of mass with age; correlation with vertebral rotation	Provides a biomechanical basis for age-related changes in curve type	[88]
Animal model (whale)	Postmortem CT and spinal morphology analysis	To test the universality of compensatory scoliosis mechanisms	Injury induced local deviation and compensatory 3D curves resembling those in humans	Scoliosis represents a universal 3D equilibrium response of the spinal system	[89]
Longitudinal observational study	169 children with early idiopathic scoliosis (Cobb < 25°, Risser 0), untreated	To investigate balancing mechanisms underlying progression and regression	Frequent changes in curve pattern; regression in 32.5% and progression in 26%	Active spinal self-balancing mechanisms exist during growth	[90]

**Table 5:** Sex differences in neurobiological and behavioral responses to stress.

Study Design/Model	Sample and Methods	Study Aim	Key Findings	Interpretation/Significance	Reference
Experimental stress-induction study	54 healthy adults (27 men, 27 women); stress-related, alcohol-related, and neutral visual stimuli; assessment of emotions, physiology (heart rate, blood pressure), and alcohol craving	To investigate sex differences in emotional and motivational responses to stress	Women exhibited greater anxiety and sadness; men showed a stronger diastolic blood pressure response; in men, alcohol craving was associated with emotional arousal	Stress is differentially integrated with emotional and reward systems in men and women	[92]
Experimental cognitive–behavioral study	45 young adults (23 women, 22 men); stress induction followed by the Balloon Analogue Risk Task (BART)	To assess the effect of stress on risk-taking behavior with respect to sex	Stress increased risk-taking in men and reduced it in women	Acute stress amplifies sexual dimorphism in decision-making strategies	[95]
EEG study (Trier Social Stress Test)	51 healthy adults; EEG recordings under stress and control conditions	To examine the effect of acute stress on frontal alpha asymmetry	Stress induced increased left-hemispheric frontal activation	Supports lateralized mechanisms of emotional regulation under stress	[98]
Population-based psychometric study	2816 adults (1566 women, 1250 men)	To examine sex differences in stress appraisal and coping styles	Women perceived stress as more intense and more frequently used emotion-focused and avoidant coping	Women appear more sensitive to stressors and preferentially oriented toward emotional regulation	[99]
Structural MRI combined with temperament assessment	100 healthy volunteers (50 women, 50 men); morphometry of the anterior cingulate cortex (ACC)	To relate ACC morphology to anxiety and harm avoidance	Right ACC was more pronounced in women and associated with anxiety	Provides a structural substrate for sex differences in stress responses	[100]
Structural MRI combined with alexithymia assessment	100 healthy adults (51 women, 49 men); TAS-20, TCI	To investigate the relationship between ACC morphology and alexithymia	In men, right ACC volume correlated more strongly with alexithymia; in women, with harm avoidance	Sex-specific neural strategies of emotional processing	[101]
Retrospective clinical–psychological study	18 individuals exposed to a shared traumatic event (firefighters); 1-year follow-up	To assess the role of coping strategies in PTSD development	Emotion-focused coping was associated with PTSD symptoms and dissociation	Cognitive processing style of trauma influences PTSD risk	[102]
Resting-state fMRI combined with psychometrics	102 healthy adults (67 women, 35 men)	To relate coping styles to brain connectivity	Emotion-avoidant coping was associated with reduced anticorrelation between the default mode network (DMN) and attention systems	Network-level mechanisms underlie individual stress responses	[103]
DTI combined with personality profiling	Healthy adults; white matter integrity analysis	To examine neural correlates of well-being and coping	Personality profiles were associated with white matter tract integrity	Stress resilience has structural neural correlates	[104]

**Table 6:** Sex differences in the development and progression of adolescent idiopathic scoliosis (AIS): morphological, biomechanical, and neurobiological evidence.

Study Design/Model	Sample and Methods	Study Aim	Key Findings	Interpretation/Significance	Reference
Neonatal MRI study	70 full-term neonates (35 boys, 35 girls); spinal morphometry	To identify congenital sex differences in the axial skeleton	Girls exhibited smaller vertebral cross-sectional area (CSA) despite comparable body size	Congenitally reduced mechanical strength of the spine in females	[108]
Pediatric MRI study	80 children aged 9–13 years (40 boys, 40 girls)	To relate vertebral CSA to lumbar lordosis	Girls had smaller CSA and greater lumbar lordosis; negative CSA–lordosis correlation	Vertebral morphology determines sagittal spinal profile	[109]
Longitudinal posture study	194 children (5–16 years); 5-year follow-up	To analyze developmental trajectories of kyphosis and lordosis	Lordosis was more pronounced in girls; no sex differences in kyphosis	Sex-specific formation of the sagittal spinal profile	[110]
Experimental hormonal–biomechanical study	20 women using combined oral contraceptives (COC) and 20 non-users; tendon ultrasound	To examine the effect of estrogen on tendon deformation	Chronically low estrogen levels reduced tendon deformation	Estrogen modulates collagen structure and tissue mechanical properties	[114]
Biochemical collagen study	23 young women (11 COC users, 12 non-users); microdialysis	To assess the effect of estradiol on collagen synthesis	Estradiol suppressed load-induced collagen synthesis	Female connective tissue exhibits reduced adaptive capacity	[115]
Animal model (pigs)	Posterior cruciate ligament fibroblasts; mechanical loading + estrogen	To investigate ligament matrix regulation	Estrogen combined with mechanical load inhibited collagen expression	Mechanistic basis of tissue vulnerability in females	[116]
Comparative metabolic study	Women (*n* = 16) and men; isotopic collagen analysis	To compare collagen synthesis	Women showed lower collagen synthesis at rest and after exercise	Slower tissue repair and remodeling in women	[117]
Cohort study of injury incidence	558 military cadets (men and women)	To assess sex differences in stress-related injuries	Higher incidence of stress fractures in women	Clinical evidence of increased biomechanical vulnerability in females	[121]
Case–control study	70 patients with idiopathic scoliosis (IS) and 58 controls; Beighton score	To assess the role of joint hypermobility in IS	Generalized joint hypermobility was more prevalent in IS, particularly in girls	Association between connective tissue laxity and IS	[122]
Longitudinal brain MRI study	387 children and adolescents	To compare brain developmental trajectories	Peak gray matter volumes occurred earlier in girls	Temporal mismatch between neural and somatic maturation	[123]
Anthropometric study	>1400 children; vertebral shape analysis	To investigate sexual dimorphism in vertebral morphology	Vertebrae in girls became more slender from age 8	Increased geometric instability of the spine	[124]
Posturographic study	43 girls of different somatotypes	To relate somatotype to balance control	Ectomorphic individuals exhibited reduced postural stability	Increased risk of progression in ectomorphic girls	[125]
Clinical posturographic study	74 girls with IS of different somatotypes	To assess postural strategies	Distinct somatotypes were associated with different instability patterns	Somatotype influences IS progression	[126]
Somatotype and joint hypermobility study	60 patients with panic disorder ± agoraphobia and 60 controls	To relate ectomorphy to generalized joint hypermobility	Ectomorphy was associated with joint hypermobility	Systemic connective tissue vulnerability	[127]
Population-based longitudinal study	1060 children (515 girls, 545 boys); 11-year follow-up	To analyze growth and posture development	Stable lordosis in females; increasing kyphosis in males	Sex-specific evolution of sagittal spinal alignment	[129]
Radiographic growth study	156 healthy children	To assess sagittal alignment and spatial orientation of individual vertebrae	During puberty, girls showed increased posterior inclination and reduced stability	Critical window for PIS progression	[130]
Biomechanical MRI study	10 men and 20 women (10 runners, 10 non-athletes)	To compare tendon adaptation	Tendon adaptation was reduced in women	Limited adaptive reserve of connective tissue	[131]
Fetal neuroanatomical study	21 fetal brains	To examine sex-related brain asymmetry	Male brains exhibited greater asymmetry	Intrauterine neuroanatomical basis of sexual dimorphism	[132]
DTI connectome study	949 children and adolescents (8–22 years; 428 males, 521 females)	To investigate sex differences in connectivity development	Females showed increased interhemispheric connectivity	Sex differences in central postural control	[133]

**Table 7:** Disturbance of the body schema as a neurocognitive mechanism: from innate representations to clinical manifestations in idiopathic scoliosis.

Model/Design	Method	Study Aim	Key Findings	Interpretation	Reference
Clinical observational study	Analysis of phantom sensations in 125 individuals with limb absence: 15 with congenital limb deficiency and 26 who underwent amputation before age 6	To assess the existence of phantom limbs in congenital defects and early amputations	Phantom limbs were reported in ≥20% of congenital cases and ≥50% of amputations before age 6; phantoms were detailed and often painful	Body schema is partially innate and genetically determined	[144]
Retrospective questionnaire study	Survey of 60 children and adolescents with limb absence (27 congenital, 33 surgical/traumatic amputations)	To examine the prevalence of phantom sensations and pain	Phantom sensations and pain were significantly more frequent after surgical amputation than congenital absence	Early nociceptive afferentation enhances body schema reorganization	[145]
Cross-sectional study	252 adults (99 with congenital amputation—34 surgically corrected later; 153 with early amputation before age 6)	To assess the effect of age at amputation on phantom phenomena	Most vivid phantom experiences occurred after amputation at ages 5–6; minimal phantoms when amputation occurred before age 5	Body schema is particularly plastic during early ontogenesis	[149]
Experimental infant study	Immediate and delayed imitation tasks in 40 six-week-old infants	To investigate early mechanisms of bodily representation	Evidence of memory-based imitation and motor optimization	Body schema emerges very early and relies on internal representations	[152]
Clinical neuropsychological study	Comparison of 24 preterm (14 boys) and 24 full-term (11 boys) children	To assess the impact of early sensorimotor deprivation on body schema	Preterm children showed deficits in holistic body processing and body schema	Adequate sensorimotor stimulation is critical for body schema development	[153]
Clinical psychometric study	Validation of BIDQ-S in adolescents with IS: phase 1—49 IS patients (37 females); phase 2—98 IS patients (75 females) and 98 controls	To assess body perception disturbances in IS	Patients with IS showed significantly greater body image distortion	IS is associated with impaired bodily self-perception	[155]
Comparative clinical study	Assessment of body awareness in 96 patients with IS and 71 controls	To relate body awareness to quality of life	Reduced body awareness correlated with pain, mental health impairment, and reduced quality of life	Body schema is a key determinant of quality of life in IS	[156]
Clinical observational study	Graphic assessment of perceived spinal curvature in 44 adolescents with IS	To examine awareness of trunk displacement	Systematic over- and underestimation of deformity magnitude	Internal representation of the body axis is disrupted	[157]
Cross-sectional clinical study	15 individuals with IS (5 males, 10 females; curve magnitude 10–25°); SRS-22, TAPS, photogrammetry	To assess body image in mild IS	Even mild IS was associated with impaired body image	Body schema disruption occurs early in IS	[158]
Comparative neuropsychological study	Patients with parietal cortex lesions	To investigate the role of the parietal lobe in spatial body representation	Dissociation between exploratory and goal-directed behavior	The parietal cortex is a key node of the body schema network	[159]
Clinical neuropsychological study	64 patients with unilateral stroke and 41 controls	To examine body schema deficits	Body schema deficits were more frequent following left-hemispheric lesions	Body schema represents a distinct cognitive system	[160]
Clinical case report	62-year-old patient with Alzheimer’s disease	To test the multilevel organization of body knowledge	Dissociation between semantic and spatial body knowledge	Body schema is not a unitary function	[141]
Neuroimaging case report	fMRI and TMS in a patient with congenital limb absence	To investigate the neural basis of aplastic phantoms	Activation of premotor and parietal cortex without primary S1/M1 involvement	Body representations can exist without peripheral input	[150]
Clinical case series	Analysis of phantom visions in congenital limb absence (11-year-old girl) and early amputations (two adults aged 23 and 50; amputation at age 5)	To describe the phenomenology of phantom experiences	Phantom experiences were as vivid as in typical adult amputees	Body schema develops independently of sensorimotor experience	[151]

**Table 8:** Conversion disorder: psychotrauma, sexual dimorphism, and neurobiological mechanisms.

Model/Design	Method	Study Aim	Key Findings	Interpretation/Pathogenetic Significance	Reference
Clinical comparative study	54 patients with conversion disorder (CD; 45 women, 9 men) vs. 50 patients with affective disorders (41 women, 9 men); trauma interviews; dissociation scales	To assess the association between childhood trauma and CD and the role of hypnotic suggestibility	CD patients reported higher rates of physical and sexual abuse; hypnotic suggestibility partially mediated the relationship between trauma and symptom severity	CD is linked to trauma exposure and dissociative processing of experience	[167]
Descriptive cross-sectional study	100 patients with dissociative disorders	To examine stressors, family environment, and coping strategies	87% were women; marked family stressors; sex differences in coping strategies	Social and familial factors amplify and maintain symptoms	[171]
Case–control study	199 patients with functional motor disorders (FMD; 149 women) and 95 controls (60 women)	To assess the association between sexual abuse and sex	Sexual abuse was associated with FMD in women but not in men	Explains higher prevalence of functional neurological disorders in women	[172]
Population-based case–control study	276 cases and 261 controls (Brazil)	To investigate the influence of sex and social factors	Female sex was associated with common mental disorders, particularly after age 30	Social factors modulate psychopathology	[173]
fMRI study	12 patients with motor CD and 14 controls	To investigate processing of negative emotions	Hyperactivation of the amygdala, periaqueductal gray, and anterior cingulate cortex; lack of habituation	Emotional hyperreactivity drives motor dysfunction	[174]
Functional neuroimaging study (SPECT)	7 patients (6 women, 1 man) with unilateral symptoms	To identify neurophysiological markers of hysterical motor deficit	Reversible contralateral hypoactivation of the thalamus and basal ganglia	Functional “blockade” of motor circuits	[175]
Voxel-based morphometry (VBM) study	23 patients of mixed sex with FND (secondary analysis in 18 women)	To link trauma, FND, and brain morphometry	In women, reduced anterior insular cortex volume was associated with greater symptom severity	The cingulo–insular network is a key node in FND	[176]
Interdisciplinary clinical study	114 patients (86 women, 28 men) with CD	To assess symptom lateralization	No consistent symptom lateralization	CD cannot be explained by simple hemispheric asymmetry	[177]
Clinical neurophysiological study	79 patients with psychogenic non-epileptic seizures (PNES) vs. 122 with epilepsy	To test the role of lateralized dysfunction	Right-hemispheric dysfunction was present in 71%	The nondominant hemisphere is involved in pathogenesis	[178]
Cohort biomarker study	149 women with non-suicidal self-injury (NSSI) and 40 controls	To identify biological markers underlying NSSI and associated psychopathology	Low oxytocin levels, reduced pain sensitivity, inflammatory markers	Somatic–affective dysregulation	[179]
Clinical adolescent study	108 adolescents with self-injury (32 boys, 76 girls)	To investigate functions of NSSI	Predominance of automatic (intrapersonal) reinforcement	Bodily symptoms serve affect regulation	[180]
Pediatric clinical case report	Two boys aged 12 and 10 years with CD	To describe CD in childhood	Early recognition improved prognosis	CD is a reversible functional disorder	[164]
Clinical case with neuroimaging	Woman with left-sided paralysis without somatosensory deficits	To investigate neural mechanisms of paralysis	Activation of the anterior cingulate cortex and right orbitofrontal cortex instead of primary motor cortex	Emotion-driven inhibition of voluntary movement	[168]

**Table 9:** Development of the “Distorting Mirror Effect”: neural, cognitive, and postural mechanisms.

Model/Design	Method	Study Aim	Key Findings	Interpretation for The “Curved Mirror Effect”	Reference
fMRI decision-making study	8 healthy volunteers (4 men, 4 women); reward-based motor decision task	To test functional heterogeneity of the dorsal anterior cingulate cortex (dACC)	dACC activation increased with reduced reward; hierarchical activation pattern (REDrew > SWITCH > CONrew)	dACC encodes discrepancies between expectations and actual outcomes	[194]
Lesion network mapping	17 focal lesions associated with delusional misidentification syndromes	To identify the network underlying Capgras syndrome	Lesions connected to left retrosplenial cortex (recognition) and right frontal cortex (belief evaluation)	Delusional misidentification arises from desynchronization between recognition and belief validation systems	[197]
Clinical case study	63-year-old man with PTSD and alcohol dependence	To investigate the role of the right hemisphere in misidentification syndromes	Right-hemispheric hypoperfusion with marked cognitive and identity distortions	Right-hemisphere dysfunction impairs filtering of anomalous internal representations	[198]
Clinical case with vestibular stimulation	Patient with somatoparaphrenic delusion	To assess the effect of vestibular stimulation on somatoparaphrenia	Cold caloric vestibular stimulation transiently reduced delusional symptoms	Vestibular input can recalibrate distorted body schema	[199]
Eye-movement experiment	8 healthy participants	To examine the influence of internal motion representation on oculomotor responses	Eye movement amplitude depended on subjective interpretation of motion	Sensorimotor responses are shaped by internal body models rather than purely reflexive mechanisms	[200]
fMRI combined with Stroop task	12 healthy right-handed participants	To examine anterior cingulate cortex (ACC) function	ACC detected conflict but did not directly resolve it	ACC functions as a mismatch detector—a “curvature sensor” of cognitive models	[201]
Comparative animal study (macaques)	9 rhesus macaques (3 with ACC lesions, 6 controls)	To investigate the role of ACC in value-based learning	Impaired maintenance of advantageous strategies despite preserved error responses	ACC is required for stable integration of experience and expectations	[202]
Unilateral electroconvulsive therapy (ECT)	16 patients with depression and schizophrenia	To investigate hemispheric organization of language and meaning	Right hemisphere initiated meaning construction; left hemisphere formalized it	Fundamentally distinct hemispheric roles in meaning generation	[203]
ECT combined with syllogistic reasoning	24 patients: study 1–14 right-handers (9 women, 5 men); study 2–10 right-handed women	To compare hemispheric reasoning styles	Left hemisphere: abstract, decontextualized; right hemisphere: contextual	Reality distortion depends on interhemispheric imbalance	[204]
Animal model (rats)	Transfer of brain extracts	To test biochemical transmission of asymmetry	Postural asymmetry transferred to recipient animals	“Curvature” can be stabilized at a molecular level	[205]
Animal model and human tissue study	Mice with idiopathic scoliosis and paraspinal muscles from patients	To investigate the role of ESR1 in muscular asymmetry	Asymmetric ESR1 inactivation induced scoliosis; raloxifene slowed progression	Peripheral asymmetry reinforces and stabilizes a distorted body axis	[206]

**Table 10:** Integrative model of adolescent idiopathic scoliosis (AIS): from brain to spine.

System Level	Normal Function	Identified Disturbances (by Sections)	Distortion Mechanism	Clinical Manifestation	Contribution to PIS
Genetic–hormonal	Regulation of growth, symmetry, and tissue adaptation	Sex-specific ESR1 expression, estrogen modulation, delayed skeletal stabilization in girls	Asymmetric hormonal sensitivity of tissues	Increased plasticity and reduced mechanical stability	Predisposition
Molecular–tissue	Balance of collagen turnover, muscle and ligament stiffness	Reduced collagen synthesis in females; asymmetry of paraspinal muscles	Differential adaptation rates to mechanical load	Micro-instability of spinal segments	Initiation of asymmetry
Postural–biomechanical	Maintenance of upright equilibrium	Age-related kyphosis–lordosis shifts, center-of-mass displacement, intrinsic rotational patterns	Fixation of asymmetric mechanical loading	Three-dimensional spinal deformation	Progression
Sensorimotor	Integration of proprioception and motor control	Impaired postural control; somatotype-dependent responses	Feedback processing errors	Unstable body axis	Maintenance of deformation
Cortical (ACC–insula–PPC)	Error monitoring and bodily awareness	dACC hypersensitivity; insular distortion of interoception	Impaired error correction	Habituation to distortion	Pattern fixation
Interhemispheric	Balance between global and local control	Dominance of right-hemispheric processing	Disturbance of the body schema	Asymmetric perception	Amplification of curvature
Cognitive–perceptual	Accurate body image	Disturbance of the body schema; self-assessment errors	“ Distorting Mirror Effect”	Under-/overestimation of deformity	Delayed diagnosis
Emotional–stress	Stress adaptation	Sex-specific stress reactivity	Stress-induced failure of corrective mechanisms	Pubertal progression	Trigger
Behavioral	Postural correction and compliance	Reduced bodily awareness	Reinforcement of maladaptive motor patterns	Lack of self-correction	Chronification

## Data Availability

No new data were created or analyzed in this study. Data sharing is not applicable to this article.

## Supplementary Materials

The following supporting information can be downloaded at: https://www.mdpi.com/article/10.3390/life16020270/s1, Table S1: Characteristics and distribution of included studies by thematic categories. Values in square brackets indicate the primary references assigned to each thematic category, while values in parentheses denote additional references that recur across multiple sections. The total number of unique studies included across all categories is *n* = 225.

## References

[1] Konieczny M.R., Senyurt H., Krauspe R. (2013). Epidemiology of Adolescent Idiopathic Scoliosis. J. Child. Orthop..

[2] Artusi C.A., Montanaro E., Tuttobene S., Romagnolo A., Zibetti M., Lopiano L. (2019). Pisa Syndrome in Parkinson’s Disease Is Associated with Specific Cognitive Alterations. Front. Neurol..

[3] Ali F., Matsumoto J.Y., Hassan A. (2018). Camptocormia. Neurol. Clin. Pract..

[4] Finsterer J., Revuelta G.J. (2014). Anterocollis and Anterocaput. Clin. Neurol. Neurosurg..

[5] Liu T., Chu W.C.W., Young G., Li K., Yeung B.H.Y., Guo L., Man G.C.W., Lam W.W.M., Wong S.T.C., Cheng J.C.Y. (2008). MR Analysis of Regional Brain Volume in Adolescent Idiopathic Scoliosis: Neurological Manifestation of a Systemic Disease. J. Magn. Reson. Imaging.

[6] Shi L., Wang D., Chu W.C.W., Burwell R.G., Freeman B.J.C., Heng P.A., Cheng J.C.Y. (2009). Volume-Based Morphometry of Brain MR Images in Adolescent Idiopathic Scoliosis and Healthy Control Subjects. Am. J. Neuroradiol..

[7] Xue C., Shi L., Hui S.C.N., Wang D., Lam T.P., Ip C.-B., Ng B.K.W., Cheng J.C.Y., Chu W.C.W. (2018). Altered White Matter Microstructure in the Corpus Callosum and Its Cerebral Interhemispheric Tracts in Adolescent Idiopathic Scoliosis: Diffusion Tensor Imaging Analysis. Am. J. Neuroradiol..

[8] Chu W.C.W., Man G.C.W., Lam W.W.M., Yeung B.H.Y., Chau W., Ng B.K.W., Lam T., Lee K., Cheng J.C.Y. (2007). A Detailed Morphologic and Functional Magnetic Resonance Imaging Study of the Craniocervical Junction in Adolescent Idiopathic Scoliosis. Spine.

[9] Lee R.K.L., Griffith J.F., Leung J.H.Y., Chu W.C.W., Lam T.P., Ng B.K.W., Cheng J.C.Y. (2015). Effect of Upright Position on Tonsillar Level in Adolescent Idiopathic Scoliosis. Eur. Radiol..

[10] Wang D., Shi L., Chu W.C.W., Burwell R.G., Cheng J.C.Y., Ahuja A.T. (2012). Abnormal Cerebral Cortical Thinning Pattern in Adolescent Girls with Idiopathic Scoliosis. Neuroimage.

[11] Barrios C., Tuñón M.T., de Salis J.A., Beguiristain J.L., Cañadell J. (1987). Scoliosis Induced by Medullary Damage: An Experimental Study in Rabbits. Spine.

[12] Maiocco B., Deeney V.F., Coulon R., Parks P.F. (1997). Adolescent Idiopathic Scoliosis and the Presence of Spinal Cord Abnormalities. Spine.

[13] Boček V., Krbec M., Vaško P., Brabec K., Pavlíková M., Štětkářová I. (2022). Alteration of Cortical but Not Spinal Inhibitory Circuits in Idiopathic Scoliosis. J. Spinal Cord. Med..

[14] Lu W.W., Hu Y., Luk K.D.K., Cheung K.M.C., Leong J.C.Y. (2002). Paraspinal Muscle Activities of Patients with Scoliosis After Spine Fusion. Spine.

[15] Wilczyński J., Karolak P. (2021). Relationship Between Electromyographic Frequency of the Erector Spinae and Location, Direction, and Number of Spinal Curvatures in Children with Scoliotic Changes. Risk Manag. Healthc. Policy.

[16] Goldberg C.J., Dowling F.E., Fogarty E.E., Moore D.P. (1995). Adolescent Idiopathic Scoliosis and Cerebral Asymmetry. Spine.

[17] Wang D., Shi L., Liu S., Hui S.C.N., Wang Y., Cheng J.C.Y., Chu W.C.W. (2013). Altered Topological Organization of Cortical Network in Adolescent Girls with Idiopathic Scoliosis. PLoS ONE.

[18] Lai B., Yi A., Zhang F., Wang S., Xin J., Li S., Yu L. (2024). Atypical Brain Lateralization for Speech Processing at the Sublexical Level in Autistic Children Revealed by FNIRS. Sci. Rep..

[19] Lee S.-B., Chae H.-W., Kwon J.-W., Sung S., Lee H.-M., Moon S.-H., Lee B.H. (2021). Is There an Association Between Psychiatric Disorders and Adolescent Idiopathic Scoliosis? A Large-Database Study. Clin. Orthop. Relat. Res..

[20] Malmqvist M., Tropp H., Lyth J., Wiréhn A.-B., Castelein R.M. (2019). Patients With Idiopathic Scoliosis Run an Increased Risk of Schizophrenia. Spine Deform..

[21] Jankovic J., Leder S., Warner D., Schwartz K. (1991). Cervical Dystonia. Neurology.

[22] Kaplan L., Aurigemma E., Sullivan T., Sidlow R. (2018). Camptocormia in an Adolescent: A Case Report and Review of the Literature. Case Rep. Psychiatry.

[23] Skidmore F., Anderson K., Fram D., Weiner W. (2007). Psychogenic Camptocormia. Mov. Disord..

[24] Pfeiffer E., von Moers A. (2000). Camptocormia in an Adolescent. J. Am. Acad. Child. Adolesc. Psychiatry.

[25] Rosen J.C., Frymoyer J.W. (1985). A Review of Camptocormia and an Unusual Case in the Female. Spine.

[26] Finsterer J., Strobl W. (2010). Presentation, Etiology, Diagnosis, and Management of Camptocormia. Eur. Neurol..

[27] Papapetropoulos S., Tuchman A., Sengun C., Russell A., Mitsi G., Singer C. (2008). Anterocollis: Clinical Features and Treatment Options. Med. Sci. Monit..

[28] Ruttiman R., Eltorai A.E.M., Daniels A.H. (2018). Etiology and Management of Spinal Deformity in Patients With Parkinson’s Disease. Int. J. Spine Surg..

[29] Miletic V. (2016). Pisa Syndrome in Parkinson’s Disease: Diagnostic and Management Challenges. J. Park. Restless Legs Syndr..

[30] Doherty K.M., van de Warrenburg B.P., Peralta M.C., Silveira-Moriyama L., Azulay J.-P., Gershanik O.S., Bloem B.R. (2011). Postural Deformities in Parkinson’s Disease. Lancet Neurol..

[31] Baik J.S., Kim J.Y., Park J.H., Han S.W., Park J.H., Lee M.S. (2009). Scoliosis in Patients with Parkinson’s Disease. J. Clin. Neurol..

[32] Pinchuk D., Dudin M., Bekshayev S., Pinchuk O. (2012). Peculiarities of Brain Functioning in Children with Adolescence Idiopathic Scoliosis (AIS) According to EEG Studies. Stud. Health Technol. Inform..

[33] Wang D., Shi L., Chu W.C.W., Paus T., Cheng J.C.Y., Heng P.A. (2009). A Comparison of Morphometric Techniques for Studying the Shape of the Corpus Callosum in Adolescent Idiopathic Scoliosis. Neuroimage.

[34] Joly O., Rousié D., Jissendi P., Rousié M., Frankó E. (2014). A New Approach to Corpus Callosum Anomalies in Idiopathic Scoliosis Using Diffusion Tensor Magnetic Resonance Imaging. Eur. Spine J..

[35] Shi L., Wang D., Hui S.C.N., Tong M.C.F., Cheng J.C.Y., Chu W.C.W. (2013). Volumetric Changes in Cerebellar Regions in Adolescent Idiopathic Scoliosis Compared with Healthy Controls. Spine J..

[36] Domenech J., García-Martí G., Martí-Bonmatí L., Barrios C., Tormos J.M., Pascual-Leone A. (2011). Abnormal Activation of the Motor Cortical Network in Idiopathic Scoliosis Demonstrated by Functional MRI. Eur. Spine J..

[37] Formaggio E., Bertuccelli M., Rubega M., Di Marco R., Cantele F., Gottardello F., De Giuseppe M., Masiero S. (2022). Brain Oscillatory Activity in Adolescent Idiopathic Scoliosis. Sci. Rep..

[38] Doménech J., Tormos J.M., Barrios C., Pascual-Leone A. (2010). Motor Cortical Hyperexcitability in Idiopathic Scoliosis: Could Focal Dystonia Be a Subclinical Etiological Factor?. Eur. Spine J..

[39] Barrios C., Arrotegui J.I. (1992). Experimental Kyphoscoliosis Induced in Rats by Selective Brain Stem Damage. Int. Orthop..

[40] Blecher R., Krief S., Galili T., Biton I.E., Stern T., Assaraf E., Levanon D., Appel E., Anekstein Y., Agar G. (2017). The Proprioceptive System Masterminds Spinal Alignment: Insight into the Mechanism of Scoliosis. Dev. Cell.

[41] Barrios C., Tunon M.T., Engstrom W., Canadell J. (1989). Paraspinal Muscle Pathology in Experimental Scoliosis. Arch. Orthop. Trauma. Surg..

[42] Cheng J.C.-Y., Chau W.-W., Guo X., Chan Y.-L. (2003). Redefining the Magnetic Resonance Imaging Reference Level for the Cerebellar Tonsil. Spine.

[43] Chiron C. (1997). The Right Brain Hemisphere Is Dominant in Human Infants. Brain.

[44] Rotenberg V., Fokin V.F., Bogolepova I.N., Gutnik B., Kobrin V.I., Shulgovsky V. (2009). Interhemispheric Asymmetry, its Function and Ontogenesis. Guide to Functional Interhemispheric Asymmetry.

[45] Bisiacchi P., Cainelli E. (2022). Structural and Functional Brain Asymmetries in the Early Phases of Life: A Scoping Review. Brain Struct. Funct..

[46] Morton B.E. (2013). Behavioral Laterality of the Brain: Support for the Binary Construct of Hemisity. Front. Psychol..

[47] Rotenberg V.S. (2008). Functional Brain Asymmetry as a Determinative Factor in the Treatment of Depression: Theoretical Implications. Prog. Neuro-Psychopharmacol. Biol. Psychiatry.

[48] Schore A.N. (2002). Dysregulation of the Right Brain: A Fundamental Mechanism of Traumatic Attachment and the Psychopathogenesis of Posttraumatic Stress Disorder. Aust. N. Z. J. Psychiatry.

[49] Weinberg I. (2000). The Prisoners of Despair: Right Hemisphere Deficiency and Suicide. Neurosci. Biobehav. Rev..

[50] Gotts S.J., Jo H.J., Wallace G.L., Saad Z.S., Cox R.W., Martin A. (2013). Two Distinct Forms of Functional Lateralization in the Human Brain. Proc. Natl. Acad. Sci. USA.

[51] Wang H., Li T., Yuan W., Zhang Z., Wei J., Qiu G., Shen J. (2019). Mental Health of Patients with Adolescent Idiopathic Scoliosis and Their Parents in China: A Cross-Sectional Survey. BMC Psychiatry.

[52] D’Agata E., Sánchez-Raya J., Bagó J. (2017). Introversion, the Prevalent Trait of Adolescents with Idiopathic Scoliosis: An Observational Study. Scoliosis Spinal Disord..

[53] Wright C.I., Williams D., Feczko E., Barrett L.F., Dickerson B.C., Schwartz C.E., Wedig M.M. (2005). Neuroanatomical Correlates of Extraversion and Neuroticism. Cereb. Cortex.

[54] Apti A., Kuru Çolak T., Akçay B., Çolak İ. (2023). Determination of Somatotypes of Children with Adolescent Idiopathic Scoliosis and Its Relationship with Scoliosis. Ann. Clin. Anal. Med..

[55] Barrios C., Cortés S., Pérez-Encinas C., Escrivá M.D., Benet I., Burgos J., Hevia E., Pizá G., Domenech P. (2011). Anthropometry and Body Composition Profile of Girls With Nonsurgically Treated Adolescent Idiopathic Scoliosis. Spine.

[56] Le Blanc R., Labelle H., Forest F., Poitras B., Rivard C.H. (1995). Possible Relationship between Idiopathic Scoliosis and Morphologic Somatotypes in Adolescent Females. Ann. Chir..

[57] LeBlanc R., Labelle H., Rivard C.-H., Poitras B. (1997). Relation Between Adolescent Idiopathic Scoliosis and Morphologic Somatotypes. Spine.

[58] Nielsen J.A., Zielinski B.A., Fletcher P.T., Alexander A.L., Lange N., Bigler E.D., Lainhart J.E., Anderson J.S. (2014). Abnormal Lateralization of Functional Connectivity between Language and Default Mode Regions in Autism. Mol. Autism.

[59] de Guibert C., Maumet C., Jannin P., Ferré J.-C., Tréguier C., Barillot C., Le Rumeur E., Allaire C., Biraben A. (2011). Abnormal Functional Lateralization and Activity of Language Brain Areas in Typical Specific Language Impairment (Developmental Dysphasia). Brain.

[60] Oh C.H., Shim Y.S., Yoon S.H., Park H., Park C.O., Lee M.S. (2013). The Psychopathological Influence of Adolescent Idiopathic Scoliosis in Korean Male: An Analysis of Multiphasic Personal Inventory Test Results. J. Korean Neurosurg. Soc..

[61] Morton B.E. (2012). Left and Right Brain-Oriented Hemisity Subjects Show Opposite Behavioral Preferences. Front. Physiol..

[62] Mitsiaki I., Thirios A., Panagouli E., Bacopoulou F., Pasparakis D., Psaltopoulou T., Sergentanis T.N., Tsitsika A. (2022). Adolescent Idiopathic Scoliosis and Mental Health Disorders: A Narrative Review of the Literature. Children.

[63] Rizzo-Sierra C.V., Leon-S M.E., Leon-Sarmiento F.E. (2012). Higher Sensory Processing Sensitivity, Introversion and Ectomorphism: New Biomarkers for Human Creativity in Developing Rural Areas. J. Neurosci. Rural. Pract..

[64] Berretz G., Wolf O.T., Güntürkün O., Ocklenburg S. (2020). Atypical Lateralization in Neurodevelopmental and Psychiatric Disorders: What Is the Role of Stress?. Cortex.

[65] Balocchini E., Chiamenti G., Lamborghini A. (2013). Adolescents: Which Risks for Their Life and Health?. J. Prev. Med. Hyg..

[66] Ernst M., Mueller S.C. (2008). The Adolescent Brain: Insights from Functional Neuroimaging Research. Dev. Neurobiol..

[67] Eiland L., Romeo R.D. (2013). Stress and the Developing Adolescent Brain. Neuroscience.

[68] Chen Y., Baram T.Z. (2016). Toward Understanding How Early-Life Stress Reprograms Cognitive and Emotional Brain Networks. Neuropsychopharmacology.

[69] Teicher M.H., Samson J.A. (2013). Childhood Maltreatment and Psychopathology: A Case for Ecophenotypic Variants as Clinically and Neurobiologically Distinct Subtypes. Am. J. Psychiatry.

[70] Pechtel P., Pizzagalli D.A. (2011). Effects of Early Life Stress on Cognitive and Affective Function: An Integrated Review of Human Literature. Psychopharmacology.

[71] Saleh A., Potter G.G., McQuoid D.R., Boyd B., Turner R., MacFall J.R., Taylor W.D. (2017). Effects of Early Life Stress on Depression, Cognitive Performance and Brain Morphology. Psychol. Med..

[72] Lenroot R.K., Giedd J.N. (2010). Sex Differences in the Adolescent Brain. Brain Cogn..

[73] Andersen S.L., Tomada A., Vincow E.S., Valente E., Polcari A., Teicher M.H. (2008). Preliminary Evidence for Sensitive Periods in the Effect of Childhood Sexual Abuse on Regional Brain Development. J. Neuropsychiatry Clin. Neurosci..

[74] Peña C.J., Kronman H.G., Walker D.M., Cates H.M., Bagot R.C., Purushothaman I., Issler O., Loh Y.-H.E., Leong T., Kiraly D.D. (2017). Early Life Stress Confers Lifelong Stress Susceptibility in Mice via Ventral Tegmental Area OTX_2_. Science.

[75] de Moraes V.S., Fernandes M., de Fátima Fernandes M.N., Gimenez L.B.H., Camargo Júnior E.B., da Silva Gherardi-Donato E.C. (2023). Relationship between Early-Life Stress and Trait Mindfulness in Adulthood: A Correlational Study. BMC Psychol..

[76] Shaparenko P.F., Krisiuk A.P., Kisil’ I.I., Goncharuk V.P. (1996). The Principle of the Spiral-like Structure of Skeletal Muscles--the Basis for Motor Optimal Performance of the Active Lower Extremity. Morfologiia.

[77] Shaparenko P.F., Pshenichnyĭ N.F. (1988). Principle of Spiral Arrangement of the Skeletal Muscles of Humans and Animals. Arkh. Anat. Gistol. Embriol..

[78] Petrova R.M., Keĭs G.D. (1981). Decussations in the Human Muscular System. Arkh. Anat. Gistol. Embriol..

[79] Janssen M.M.A., Kouwenhoven J.-W.M., Castelein R.M. (2010). The Role of Posteriorly Directed Shear Loads Acting on a Pre-Rotated Growing Spine: A Hypothesis on the Pathogenesis of Idiopathic Scoliosis. Stud. Health Technol. Inform..

[80] Lincoln T.L. (2007). Infantile Idiopathic Scoliosis. Am. J. Orthop..

[81] Yang Z., Li M. (2011). There May Be a Same Mechanism of the Left–Right Handedness and Left–Right Convex Curve Pattern of Adolescent Idiopathic Scoliosis. Med. Hypotheses.

[82] Sarnadsky V. (2012). Gender and Age Features of Postural Disorders in the Sagittal Plane in Children and Adolescents on Evidence of Computer Optical Topography. Hir. Pozvonočnika.

[83] Janssen M.M.A., Kouwenhoven J.-W.M., Schlösser T.P.C., Viergever M.A., Bartels L.W., Castelein R.M., Vincken K.L. (2011). Analysis of Preexistent Vertebral Rotation in the Normal Infantile, Juvenile, and Adolescent Spine. Spine.

[84] Kouwenhoven J.-W.M., Vincken K.L., Bartels L.W., Castelein R.M. (2006). Analysis of Preexistent Vertebral Rotation in the Normal Spine. Spine.

[85] Janssen M.M.A., Vincken K.L., Kemp B., Obradov M., de Kleuver M., Viergever M.A., Castelein R.M., Bartels L.W. (2010). Pre-Existent Vertebral Rotation in the Human Spine Is Influenced by Body Position. Eur. Spine J..

[86] Schlösser T.P.C., Semple T., Carr S.B., Padley S., Loebinger M.R., Hogg C., Castelein R.M. (2017). Scoliosis Convexity and Organ Anatomy Are Related. Eur. Spine J..

[87] Tallroth K., Lohman M., Heliövaara M., Aromaa A., Knekt P., Standertskjöld-Nordenstam C.-G. (2009). Dextrocardia and Coronal Alignment of Thoracic Curve: A Population Study. Eur. Spine J..

[88] de Reuver S., Brink R.C., Homans J.F., Kruyt M.C., van Stralen M., Schlösser T.P.C., Castelein R.M. (2019). The Changing Position of the Center of Mass of the Thorax During Growth in Relation to Pre-Existent Vertebral Rotation. Spine.

[89] de Reuver S., IJsseldijk L.L., Homans J.F., Willems D.S., Veraa S., van Stralen M., Kik M.J.L., Kruyt M.C., Gröne A., Castelein R.M. (2021). What a Stranded Whale with Scoliosis Can Teach Us about Human Idiopathic Scoliosis. Sci. Rep..

[90] Modi H.N., Suh S.-W., Yang J.-H., Hong J.-Y., Venkatesh K., Muzaffar N. (2010). Spontaneous Regression of Curve in Immature Idiopathic Scoliosis—Does Spinal Column Play a Role to Balance? An Observation with Literature Review. J. Orthop. Surg. Res..

[91] Ordaz S., Luna B. (2012). Sex Differences in Physiological Reactivity to Acute Psychosocial Stress in Adolescence. Psychoneuroendocrinology.

[92] Chaplin T.M., Hong K., Bergquist K., Sinha R. (2008). Gender Differences in Response to Emotional Stress: An Assessment Across Subjective, Behavioral, and Physiological Domains and Relations to Alcohol Craving. Alcohol. Clin. Exp. Res..

[93] Kajantie E., Phillips D.I.W. (2006). The Effects of Sex and Hormonal Status on the Physiological Response to Acute Psychosocial Stress. Psychoneuroendocrinology.

[94] Byrnes J.P., Miller D.C., Schafer W.D. (1999). Gender Differences in Risk Taking: A Meta-Analysis. Psychol. Bull..

[95] Lighthall N.R., Mather M., Gorlick M.A. (2009). Acute Stress Increases Sex Differences in Risk Seeking in the Balloon Analogue Risk Task. PLoS ONE.

[96] Rotenberg V.S. (2009). Search Activity Concept: Relationship between Behavior, Health and Brain Functions. Act. Nerv. Super..

[97] Becker J.B., Monteggia L.M., Perrot-Sinal T.S., Romeo R.D., Taylor J.R., Yehuda R., Bale T.L. (2007). Stress and Disease: Is Being Female a Predisposing Factor?. J. Neurosci..

[98] Berretz G., Packheiser J., Wolf O.T., Ocklenburg S. (2022). Acute Stress Increases Left Hemispheric Activity Measured via Changes in Frontal Alpha Asymmetries. iScience.

[99] Matud M.P. (2004). Gender Differences in Stress and Coping Styles. Pers. Individ. Dif..

[100] Pujol J., López A., Deus J., Cardoner N., Vallejo J., Capdevila A., Paus T. (2002). Anatomical Variability of the Anterior Cingulate Gyrus and Basic Dimensions of Human Personality. Neuroimage.

[101] Gündel H., López-Sala A., Ceballos-Baumann A.O., Deus J., Cardoner N., Marten-Mittag B., Soriano-Mas C., Pujol J. (2004). Alexithymia Correlates With the Size of the Right Anterior Cingulate. Psychosom. Med..

[102] Brousse, Arnaud B., Durand-Roger, Geneste J., Zaplana F., Bourguet D., Blanc O. (2011). Management of Traumatic Events: Influence of Emotion-Centered Coping Strategies on the Occurrence of Dissociation and Post-Traumatic Stress Disorder. Neuropsychiatr. Dis. Treat..

[103] Santarnecchi E., Sprugnoli G., Tatti E., Mencarelli L., Neri F., Momi D., Di Lorenzo G., Pascual-Leone A., Rossi S., Rossi A. (2018). Brain Functional Connectivity Correlates of Coping Styles. Cogn. Affect. Behav. Neurosci..

[104] Kotikalapudi R., Dricu M., Moser D.A., Aue T. (2022). Whole-Brain White Matter Correlates of Personality Profiles Predictive of Subjective Well-Being. Sci. Rep..

[105] Castelein R.M., Pasha S., Cheng J.C., Dubousset J. (2020). Idiopathic Scoliosis as a Rotatory Decompensation of the Spine. J. Bone Miner. Res..

[106] Gilsanz V., Wren T.A.L., Ponrartana S., Mora S., Rosen C.J. (2018). Sexual Dimorphism and the Origins of Human Spinal Health. Endocr. Rev..

[107] Wren T.A.L., Ponrartana S., Gilsanz V. (2017). Vertebral Cross-Sectional Area: An Orphan Phenotype with Potential Implications for Female Spinal Health. Osteoporos. Int..

[108] Ponrartana S., Aggabao P.C., Dharmavaram N.L., Fisher C.L., Friedlich P., Devaskar S.U., Gilsanz V. (2015). Sexual Dimorphism in Newborn Vertebrae and Its Potential Implications. J. Pediatr..

[109] Wren T.A.L., Aggabao P.C., Poorghasamians E., Chavez T.A., Ponrartana S., Gilsanz V. (2017). Association between Vertebral Cross-Sectional Area and Lumbar Lordosis Angle in Adolescents. PLoS ONE.

[110] Gardner A., Berryman F., Pynsent P. (2018). The Development of Kyphosis and Lordosis in the Growing Spine. Spine.

[111] Chidi-Ogbolu N., Baar K. (2019). Effect of Estrogen on Musculoskeletal Performance and Injury Risk. Front. Physiol..

[112] Magnusson S.P., Hansen M., Langberg H., Miller B., Haraldsson B., Kjoeller Westh E., Koskinen S., Aagaard P., Kjær M. (2007). The Adaptability of Tendon to Loading Differs in Men and Women. Int. J. Exp. Pathol..

[113] Kjær M., Hansen M. (2008). The Mystery of Female Connective Tissue. J. Appl. Physiol..

[114] Bryant A.L., Clark R.A., Bartold S., Murphy A., Bennell K.L., Hohmann E., Marshall-Gradisnik S., Payne C., Crossley K.M. (2008). Effects of Estrogen on the Mechanical Behavior of the Human Achilles Tendon in Vivo. J. Appl. Physiol..

[115] Hansen M., Koskinen S.O., Petersen S.G., Doessing S., Frystyk J., Flyvbjerg A., Westh E., Magnusson S.P., Kjaer M., Langberg H. (2008). Ethinyl Oestradiol Administration in Women Suppresses Synthesis of Collagen in Tendon in Response to Exercise. J. Physiol..

[116] Lee C.-Y., Liu X., Smith C.L., Zhang X., Hsu H.-C., Wang D.-Y., Luo Z.-P. (2004). The Combined Regulation of Estrogen and Cyclic Tension on Fibroblast Biosynthesis Derived from Anterior Cruciate Ligament. Matrix Biol..

[117] Miller B.F., Hansen M., Olesen J.L., Schwarz P., Babraj J.A., Smith K., Rennie M.J., Kjaer M. (2007). Tendon Collagen Synthesis at Rest and after Exercise in Women. J. Appl. Physiol..

[118] Remvig L., Jensen D.V., Ward R.C. (2007). Epidemiology of General Joint Hypermobility and Basis for the Proposed Criteria for Benign Joint Hypermobility Syndrome: Review of the Literature. J. Rheumatol..

[119] Sharp H.E.C., Critchley H.D., Eccles J.A. (2021). Connecting Brain and Body: Transdiagnostic Relevance of Connective Tissue Variants to Neuropsychiatric Symptom Expression. World J. Psychiatry.

[120] Pailhez G., Castaño J., Rosado S., Ballester M.D.M., Vendrell C., Mallorquí-Bagué N., Baeza-Velasco C., Bulbena A. (2015). Joint Hypermobility, Anxiety, and Psychosomatics—The New Neuroconnective Phenotype. A Fresh Look at Anxiety Disorders.

[121] Bijur P.E. (1997). Comparison of Injury During Cadet Basic Training by Gender. Arch. Pediatr. Adolesc. Med..

[122] Czaprowski D., Kotwicki T., Pawłowska P., Stoliński L. (2011). Joint Hypermobility in Children with Idiopathic Scoliosis: SOSORT Award 2011 Winner. Scoliosis.

[123] Lenroot R.K., Gogtay N., Greenstein D.K., Wells E.M., Wallace G.L., Clasen L.S., Blumenthal J.D., Lerch J., Zijdenbos A.P., Evans A.C. (2007). Sexual Dimorphism of Brain Developmental Trajectories during Childhood and Adolescence. Neuroimage.

[124] Taylor J.R., Twomey L.T. (1984). Sexual Dimorphism in Human Vertebral Body Shape. J. Anat..

[125] Allard P., Nault M.-L., Hinse S., LeBlanc R., Labelle H. (2001). Relationship between Morphologic Somatotypes and Standing Posture Equilibrium. Ann. Hum. Biol..

[126] Allard P., Chavet P., Barbier F., Gatto L., Labelle H., Sadeghi H. (2004). Effect of Body Morphology on Standing Balance in Adolescent Idiopathic Scoliosis. Am. J. Phys. Med. Rehabil..

[127] Pailhez G., Rosado S., Baeza-Velasco C., Bulbena A. (2014). Ectomorphic Somatotype and Joint Hypermobility Are Linked in Panic and Agoraphobic Patients: A Case-Control Study. Int. J. Psychiatry Clin. Pract..

[128] Castelein R.M., van Dieën J.H., Smit T.H. (2005). The Role of Dorsal Shear Forces in the Pathogenesis of Adolescent Idiopathic Scoliosis—A Hypothesis. Med. Hypotheses.

[129] Poussa M.S., Heliövaara M.M., Seitsamo J.T., Könönen M.H., Hurmerinta K.A., Nissinen M.J. (2005). Development of Spinal Posture in a Cohort of Children from the Age of 11 to 22 Years. Eur. Spine J..

[130] Schlösser T.P.C., Vincken K.L., Rogers K., Castelein R.M., Shah S.A. (2015). Natural Sagittal Spino-Pelvic Alignment in Boys and Girls before, at and after the Adolescent Growth Spurt. Eur. Spine J..

[131] Westh E., Kongsgaard M., Bojsen-Moller J., Aagaard P., Hansen M., Kjaer M., Magnusson S.P. (2008). Effect of Habitual Exercise on the Structural and Mechanical Properties of Human Tendon, in Vivo, in Men and Women. Scand. J. Med. Sci. Sports.

[132] De Lacoste M.C., Horvath D.S., Woodward D.J. (1991). Possible Sex Differences in the Developing Human Fetal Brain. J. Clin. Exp. Neuropsychol..

[133] Ingalhalikar M., Smith A., Parker D., Satterthwaite T.D., Elliott M.A., Ruparel K., Hakonarson H., Gur R.E., Gur R.C., Verma R. (2014). Sex Differences in the Structural Connectome of the Human Brain. Proc. Natl. Acad. Sci. USA.

[134] Anna G., Polunina E.A.B. (2017). Neuroanatomic Differences of the Brain in Males and Females. Ann. Clin. Exp. Neurol..

[135] Cardinali L., Brozzoli C., Farnè A. (2009). Peripersonal Space and Body Schema: Two Labels for the Same Concept?. Brain Topogr..

[136] Sattin D., Parma C., Lunetta C., Zulueta A., Lanzone J., Giani L., Vassallo M., Picozzi M., Parati E.A. (2023). An Overview of the Body Schema and Body Image: Theoretical Models, Methodological Settings and Pitfalls for Rehabilitation of Persons with Neurological Disorders. Brain Sci..

[137] Tsakiris M. (2010). My Body in the Brain: A Neurocognitive Model of Body-Ownership. Neuropsychologia.

[138] Holmes N.P., Spence C. (2004). The Body Schema and Multisensory Representation(s) of Peripersonal Space. Cogn. Process..

[139] Naito E., Morita T., Amemiya K. (2016). Body Representations in the Human Brain Revealed by Kinesthetic Illusions and Their Essential Contributions to Motor Control and Corporeal Awareness. Neurosci. Res..

[140] Assaiante C., Barlaam F., Cignetti F., Vaugoyeau M. (2014). Body Schema Building during Childhood and Adolescence: A Neurosensory Approach. Neurophysiol. Clin. Neurophysiol..

[141] Sirigu A., Grafman J., Bressler K., Sunderland T. (1991). Multiple Representations Contribute to Body Knowledge Processing. Brain.

[142] Li K., Malhotra P.A. (2015). Spatial Neglect. Pract. Neurol..

[143] Melzack R. (1990). Phantom Limbs and the Concept of a Neuromatrix. Trends Neurosci..

[144] Melzack R., Israel R., Lacroix R., Schultz G. (1997). Phantom Limbs in People with Congenital Limb Deficiency or Amputation in Early Childhood. Brain.

[145] Wilkins K.L., McGrath P.J., Finley A.G., Katz J. (1998). Phantom Limb Sensations and Phantom Limb Pain in Child and Adolescent Amputees. Pain.

[146] Karnath H.-O., Rorden C. (2012). The Anatomy of Spatial Neglect. Neuropsychologia.

[147] Vallar G. (1997). Spatial Frames of Reference and Somatosensory Processing: A Neuropsychological Perspective. Philos. Trans. R. Soc. London Ser. B Biol. Sci..

[148] Devinsky O. (2000). Right Cerebral Hemisphere Dominance for a Sense of Corporeal and Emotional Self. Epilepsy Behav..

[149] Diers M., Fuchs X., Bekrater-Bodmann R., Flor H. (2023). Prevalence of Phantom Phenomena in Congenital and Early-Life Amputees. J. Pain.

[150] Brugger P., Kollias S.S., Müri R.M., Crelier G., Hepp-Reymond M.-C., Regard M. (2000). Beyond Re-Membering: Phantom Sensations of Congenitally Absent Limbs. Proc. Natl. Acad. Sci. USA.

[151] Poeck K. (1964). Phantoms Following Amputation in Early Childhood and in Congenital Absence of Limbs. Cortex.

[152] Meltzoff A.N., Keith Moore M. (1994). Imitation, Memory, and the Representation of Persons. Infant. Behav. Dev..

[153] Butti N., Montirosso R., Giusti L., Borgatti R., Urgesi C. (2020). Premature Birth Affects Visual Body Representation and Body Schema in Preterm Children. Brain Cogn..

[154] Bertuccelli M., Cantele F., Masiero S. (2023). Body Image and Body Schema in Adolescents with Idiopathic Scoliosis: A Scoping Review. Adolesc. Res. Rev..

[155] Auerbach J.D., Lonner B.S., Crerand C.E., Shah S.A., Flynn J.M., Bastrom T., Penn P., Ahn J., Toombs C., Bharucha N. (2014). Body Image in Patients with Adolescent Idiopathic Scoliosis. J. Bone Jt. Surg..

[156] Yagci G., Karatel M., Yakut Y. (2020). Body Awareness and Its Relation to Quality of Life in Individuals with Idiopathic Scoliosis. Percept. Mot. Ski..

[157] Picelli A., Negrini S., Zenorini A., Iosa M., Paolucci S., Smania N. (2016). Do Adolescents with Idiopathic Scoliosis Have Body Schema Disorders? A Cross-Sectional Study. J. Back. Musculoskelet. Rehabil..

[158] Belli G., Toselli S., Latessa P.M., Mauro M. (2022). Evaluation of Self-Perceived Body Image in Adolescents with Mild Idiopathic Scoliosis. Eur. J. Investig. Health Psychol. Educ..

[159] Karnath H. (1997). Spatial Orientation and the Representation of Space with Parietal Lobe Lesions. Philos. Trans. R. Soc. London Ser. B Biol. Sci..

[160] Raimo S., Boccia M., Di Vita A., Iona T., Cropano M., Ammendolia A., Colao R., Angelillo V., Maiorino A., Guariglia C. (2022). Body Representation Alterations in Patients with Unilateral Brain Damage. J. Int. Neuropsychol. Soc..

[161] Burwell R.G., Freeman B.J.C., Dangerfield P.H., Aujla R.K., Cole A.A., Kirby A.S., Polak F., Pratt R.K., Webb J.K., Moulton A. (2006). Etiologic Theories of Idiopathic Scoliosis: Neurodevelopmental Concept of Maturational Delay of the CNS Body Schema (“body-in-the-Brain”). Stud. Health Technol. Inform..

[162] Anderson-Barnes V.C., McAuliffe C., Swanberg K.M., Tsao J.W. (2009). Phantom Limb Pain—A Phenomenon of Proprioceptive Memory?. Med. Hypotheses.

[163] Owens C., Dein S. (2006). Conversion Disorder: The Modern Hysteria. Adv. Psychiatr. Treat..

[164] Leary P.M. (2003). Conversion Disorder in Childhood-Diagnosed Too Late, Investigated Too Much?. J. R. Soc. Med..

[165] Kumar S. (2004). Conversion Disorder in Childhood. J. R. Soc. Med..

[166] Ludwig L., Pasman J.A., Nicholson T., Aybek S., David A.S., Tuck S., Kanaan R.A., Roelofs K., Carson A., Stone J. (2018). Stressful Life Events and Maltreatment in Conversion (Functional Neurological) Disorder: Systematic Review and Meta-Analysis of Case-Control Studies. Lancet Psychiatry.

[167] Roelofs K., Keijsers G.P.J., Hoogduin K.A.L., Näring G.W.B., Moene F.C. (2002). Childhood Abuse in Patients With Conversion Disorder. Am. J. Psychiatry.

[168] Marshall J.C., Halligan P.W., Fink G.R., Wade D.T., Frackowiak R.S. (1997). The Functional Anatomy of a Hysterical Paralysis. Cognition.

[169] McLoughlin C., Hoeritzauer I., Cabreira V., Aybek S., Adams C., Alty J., Ball H.A., Baker J., Bullock K., Burness C. (2023). Functional Neurological Disorder Is a Feminist Issue. J. Neurol. Neurosurg. Psychiatry.

[170] Jenkins R. (1985). Women and Minor Psychiatric Morbidity. J. R. Soc. Med..

[171] Mohammad Y., Kumar R., Sinha N., Kumar P. (2023). A Study of Stressors, Family Environment, Coping Patterns, and Family Burden in Persons with Dissociative Disorder. Ind. Psychiatry J..

[172] Kletenik I., Sillau S.H., Isfahani S.A., LaFaver K., Hallett M., Berman B.D. (2020). Gender as a Risk Factor for Functional Movement Disorders: The Role of Sexual Abuse. Mov. Disord. Clin. Pract..

[173] Coutinho E.D.S.F., De Almeida Filho N., De Jesus Mari J., Rodrigues L.C. (1999). Gender and Minor Psychiatric Morbidity: Results of a Case-Control Study in a Developing Country. Int. J. Psychiatry Med..

[174] Aybek S., Nicholson T.R., O’Daly O., Zelaya F., Kanaan R.A., David A.S. (2015). Emotion-Motion Interactions in Conversion Disorder: An FMRI Study. PLoS ONE.

[175] Vuilleumier P. (2001). Functional Neuroanatomical Correlates of Hysterical Sensorimotor Loss. Brain.

[176] Perez D.L., Matin N., Barsky A., Costumero-Ramos V., Makaretz S.J., Young S.S., Sepulcre J., LaFrance W.C., Keshavan M.S., Dickerson B.C. (2017). Cingulo-Insular Structural Alterations Associated with Psychogenic Symptoms, Childhood Abuse and PTSD in Functional Neurological Disorders. J. Neurol. Neurosurg. Psychiatry.

[177] Roelofs K., Näring G.W., Moene F.C., Hoogduin C.A. (2000). The Question of Symptom Lateralization in Conversion Disorder. J. Psychosom. Res..

[178] Devinsky O., Mesad S., Alper K. (2001). Nondominant Hemisphere Lesions and Conversion Nonepileptic Seizures. J. Neuropsychiatry Clin. Neurosci..

[179] Mürner-Lavanchy I., Koenig J., Reichl C., Josi J., Cavelti M., Kaess M. (2024). The Quest for a Biological Phenotype of Adolescent Non-Suicidal Self-Injury: A Machine-Learning Approach. Transl. Psychiatry.

[180] Nock M.K., Prinstein M.J. (2004). A Functional Approach to the Assessment of Self-Mutilative Behavior. J. Consult. Clin. Psychol..

[181] Black D.N., Seritan A.L., Taber K.H., Hurley R.A. (2004). Conversion Hysteria: Lessons From Functional Imaging. J. Neuropsychiatry Clin. Neurosci..

[182] Harvey S.B., Stanton B.R., David A.S. (2006). Conversion Disorder: Towards a Neurobiological Understanding. Neuropsychiatr. Dis. Treat..

[183] Ospina J.P., Jalilianhasanpour R., Perez D.L. (2019). The Role of the Anterior and Midcingulate Cortex in the Neurobiology of Functional Neurologic Disorder. Handb. Clin. Neurol..

[184] Stone J. (2002). Are Functional Motor and Sensory Symptoms Really More Frequent on the Left? A Systematic Review. J. Neurol. Neurosurg. Psychiatry.

[185] Bègue I., Adams C., Stone J., Perez D.L. (2019). Structural Alterations in Functional Neurological Disorder and Related Conditions: A Software and Hardware Problem?. NeuroImage Clin..

[186] Devinsky O., Morrell M.J., Vogt B.A. (1995). Contributions of Anterior Cingulate Cortex to Behaviour. Brain.

[187] Yücel M., Wood S.J., Fornito A., Riffkin J., Velakoulis D., Pantelis C. (2003). Anterior Cingulate Dysfunction: Implications for Psychiatric Disorders?. J. Psychiatry Neurosci..

[188] Turner B.O., Marinsek N., Ryhal E., Miller M.B. (2015). Hemispheric Lateralization in Reasoning. Ann. N. Y. Acad. Sci..

[189] Marinsek N., Turner B.O., Gazzaniga M., Miller M.B. (2014). Divergent Hemispheric Reasoning Strategies: Reducing Uncertainty versus Resolving Inconsistency. Front. Hum. Neurosci..

[190] Goel V. (2015). Indeterminacy Tolerance as a Basis of Hemispheric Asymmetry within Prefrontal Cortex. Front. Hum. Neurosci..

[191] Devinsky O. (2009). Delusional Misidentifications and Duplications. Neurology.

[192] Reber J., Tranel D. (2017). Sex Differences in the Functional Lateralization of Emotion and Decision Making in the Human Brain. J. Neurosci. Res..

[193] Clairis N., Lopez-Persem A. (2023). Debates on the Dorsomedial Prefrontal/Dorsal Anterior Cingulate Cortex: Insights for Future Research. Brain.

[194] Bush G., Vogt B.A., Holmes J., Dale A.M., Greve D., Jenike M.A., Rosen B.R. (2002). Dorsal Anterior Cingulate Cortex: A Role in Reward-Based Decision Making. Proc. Natl. Acad. Sci. USA.

[195] Gurin L., Blum S. (2017). Delusions and the Right Hemisphere: A Review of the Case for the Right Hemisphere as a Mediator of Reality-Based Belief. J. Neuropsychiatry Clin. Neurosci..

[196] Ellis H.D. (1994). The Role of the Right Hemisphere in the Capgras Delusion. Psychopathology.

[197] Darby R.R., Laganiere S., Pascual-Leone A., Prasad S., Fox M.D. (2017). Finding the Imposter: Brain Connectivity of Lesions Causing Delusional Misidentifications. Brain.

[198] Thode K.I., Faber R.A., Chaudhuri T.K. (2012). Delusional Misidentification Syndrome: Right-Hemisphere Findings on SPECT. J. Neuropsychiatry Clin. Neurosci..

[199] Bisiach E., Rusconi M.L., Vallar G. (1991). Remission of Somatoparaphrenic Delusion through Vestibular Stimulation. Neuropsychologia.

[200] Gurfinkel V.S., Levik Y.S. (1993). The Suppression of Cervico-Ocular Response by the Haptokinetic Information about the Contact with a Rigid, Immobile Object. Exp. Brain Res..

[201] Carter C.S., Macdonald A.M., Botvinick M., Ross L.L., Stenger V.A., Noll D., Cohen J.D. (2000). Parsing Executive Processes: Strategic vs. Evaluative Functions of the Anterior Cingulate Cortex. Proc. Natl. Acad. Sci. USA.

[202] Kennerley S.W., Walton M.E., Behrens T.E.J., Buckley M.J., Rushworth M.F.S. (2006). Optimal Decision Making and the Anterior Cingulate Cortex. Nat. Neurosci..

[203] Chernigovskaya T.V., Deglin V.L. (1986). Brain Functional Asymmetry and Neural Organization of Linguistic Competence. Brain Lang..

[204] Deglin V.L., Kinsbourne M. (1996). Divergent Thinking Styles of the Hemispheres: How Syllogisms Are Solved during Transitory Hemisphere Suppression. Brain Cogn..

[205] Vartanian G.A., Balabanov I.V. (1978). Induction of Postural Asymmetry in an Intact Recipient with an Extract from the Brain of a Donor with Such a Syndrome. Biull. Eksp. Biol. Med..

[206] Shao X., Fu X., Yang J., Sui W., Li S., Yang W., Lin X., Zhang Y., Jia M., Liu H. (2023). The Asymmetrical ESR1 Signaling in Muscle Progenitor Cells Determines the Progression of Adolescent Idiopathic Scoliosis. Cell Discov..

[207] Noll-Hussong M., Holzapfel S., Pokorny D., Herberger S. (2014). Caloric Vestibular Stimulation as a Treatment for Conversion Disorder: A Case Report and Medical Hypothesis. Front. Psychiatry.

[208] Sturman D.A., Moghaddam B. (2011). The Neurobiology of Adolescence: Changes in Brain Architecture, Functional Dynamics, and Behavioral Tendencies. Neurosci. Biobehav. Rev..

[209] Verma R., Balhara Y.S., Gupta C. (2011). Gender Differences in Stress Response: Role of Developmental and Biological Determinants. Ind. Psychiatry J..

[210] Horwitz A.G., Hill R.M., King C.A. (2011). Specific Coping Behaviors in Relation to Adolescent Depression and Suicidal Ideation. J. Adolesc..

[211] Merikangas K.R., He J., Burstein M., Swanson S.A., Avenevoli S., Cui L., Benjet C., Georgiades K., Swendsen J. (2010). Lifetime Prevalence of Mental Disorders in U.S. Adolescents: Results from the National Comorbidity Survey Replication–Adolescent Supplement (NCS-A). J. Am. Acad. Child. Adolesc. Psychiatry.

[212] Diana P., Esposito S. (2023). A Gender-Based Point of View in Pediatric Neurology. J. Pers. Med..

[213] McIlwrick S., Rechenberg A., Matthes M., Burgstaller J., Schwarzbauer T., Chen A., Touma C. (2016). Genetic Predisposition for High Stress Reactivity Amplifies Effects of Early-Life Adversity. Psychoneuroendocrinology.

[214] Schultz A.B., Sörensen S.-E., Andersson G.B.J. (1984). Measurements of Spine Morphology in Children, Ages 10–16. Spine.

[215] Wang W., Wang Z., Liu Z., Zhu Z., Zhu F., Sun X., Lam T.P., Cheng J.C., Qiu Y. (2015). Are There Gender Differences in Sagittal Spinal Pelvic Inclination before and after the Adolescent Pubertal Growth Spurt?. Eur. Spine J..

[216] Bastir M., Higuero A., Ríos L., García Martínez D. (2014). Three-dimensional Analysis of Sexual Dimorphism in Human Thoracic Vertebrae: Implications for the Respiratory System and Spine Morphology. Am. J. Phys. Anthropol..

[217] Kouwenhoven J.-W.M., Smit T.H., van der Veen A.J., Kingma I., van Dieën J.H., Castelein R.M. (2007). Effects of Dorsal Versus Ventral Shear Loads on the Rotational Stability of the Thoracic Spine. Spine.

[218] Bulbena A., Pailhez G., Bulbena-Cabré A., Mallorquí-Bagué N., Baeza-Velasco C. (2015). Joint Hypermobility, Anxiety and Psychosomatics: Two and a Half Decades of Progress Toward a New Phenotype. Adv. Psychosom. Med..

[219] Bulbena A., Martín-Santos R., Porta M., Duró J.C., Gago J., Sangorrín J., Gratacós M. (1996). Somatotype in Panic Patients. Anxiety.

[220] Möllmann A., Heinrichs N., Herwig A. (2024). A Conceptual Framework on Body Representations and Their Relevance for Mental Disorders. Front. Psychol..

[221] Axelrod S., Noonan M., Atanacio B. (1980). On the Laterality of Psychogenic Somatic Symptoms. J. Nerv. Ment. Dis..

[222] Lahutsina A., Spaniel F., Mrzilkova J., Morozova A., Brabec M., Musil V., Zach P. (2022). Morphology of Anterior Cingulate Cortex and Its Relation to Schizophrenia. J. Clin. Med..

[223] Stevens F.L., Hurley R.A., Taber K.H. (2011). Anterior Cingulate Cortex: Unique Role in Cognition and Emotion. J. Neuropsychiatry Clin. Neurosci..

[224] Bresin K., Gordon K.H. (2013). Endogenous Opioids and Nonsuicidal Self-Injury: A Mechanism of Affect Regulation. Neurosci. Biobehav. Rev..

[225] Laura B., Maisto D., Pezzulo G. (2023). Modeling and Controlling the Body in Maladaptive Ways: An Active Inference Perspective on Non-Suicidal Self-Injury Behaviors. Neurosci. Conscious..

