# Supplementary material for: Idiopathic Scoliosis as a Conversion Reaction to Stress with the Neural Effect of a “Distorting Mirror”
Source: Life (Basel). 2026 Feb 4;16(2):270. doi: 10.3390/life16020270 (PMC12941543; doi:10.3390/life16020270)
Supplement: Supplementary file 1 [file life-16-00270-s001.zip › life-4100263-supplementary.pdf]

Table 1. Characteristics and distribution of included studies by thematic categories. Values in square brackets indicate the primary references assigned to each thematic category, while values in parentheses denote additional references that recur across multiple sections. The total number of unique studies included across all categories is n = 225.

Section	Category	Number	List
Introduction	Article	[3], ([13])	[15,16,17], ([2,5,6,7,8,9,10,11,13,14, ,18,19,20])
	Review	[1], ([2])	[1], ([3,4])
	Case Reports	[1]	[12]
Leading Role of the Central Nervous System in the Development of AIS	Article	[25]	[2,5,6,7,8,9,10,11,13,14,20,21,27, 31,32,33,34,35,36,37,38,39,40,41 ,42]
	Review	[6]	[3,4,26,28,29,30]
	Case Reports	[4]	[22,23,24,25]
Brain Lateralization	Article	[15], ([1])	[18,19,43,50, 51,52,53,55,56,57,58,60,61,63,64], ([20])
	Review	[8]	[45,46,47,48,49,54,59,62]
	Chapter of a book	[1]	[44]
Stress as a Trigger for the Development of AIS	Article	[4]	[71,73,74,75]
	Review	[7], ([1])	[65,66,67,68,69,70,72], ([48])
Three-Plane Spinal Deformity	Article	[9]	[82,83,84,85,86,87,88,89,90]
	Review	[1]	[80]
	Hypothesis	[5]	[76,77,78,79,81]
Sex Differences in Stress Response	Article	[9]	[92,95,98,99,100,101,102,103,104]
	Review	[5]	[91,93,94,96,97]
Sex Differences in the Development and Progression of AIS	Article	[19]	[108,109,110,114,115,116,117,121,122,123,124,125,126,127, 129,130,131,133,134]
	Review	[7], ([1])	[106,107,111,112,118,119,132],([72])
	Editorial	[1]	[113]
	Open access peer-reviewed chapter	[1]	[120]

	Hypothesis	[2]	[105,128]
Disturbance of the Body Schema	Article	[11]	[144,145, ,149,152,153,155,156,157,158,161,162]
	Review	[11]	[135,136,137,138,139,140,142,143, 146,147,154]
	Case Reports	[3]	[141,150,151]
	Hypothesis	[3]	[148,159,160]
Conversion Disorder	Article	[11]	[167,171,172,173,177,178,179, 181,182,186,187]
	Review	[10], ([1])	[163,166,169,174,175,176, 180,183,184,185],([49])
	Case Reports	[2]	[164,168]
	Comment	[1]	[165]
	Editorial	[1]	[170]
Development of the "Distorting Mirror Effect"	Article	[9]	[194,197,201,202,203,204,205,206,207]
	Review	[8]	[188,189,190,191,192,193,195,196]
	Case Reports	[2]	[198,200]
	Hypothesis	[1]	[199]
Discussion	Article	[12], ([62])	[209, 210, 211,213, 214, 215,216,217, 219, 225, 222,223], ([37,52,55,56,58,65,71,73,74,75,76,77,78,79,83,84,85,89,90, 92,95,100,102,103, 105,110, 121,122,123, 124,125,126,127, 128, 129,130,132,133,134,144,145,146,149,152,153, 155,156,157,158,167,171,172,173,177,179, 180,181,182,186,187, 197,205])
	Review	[6],([34])	[208, 212, 218, 220, 221, 224], ([48,49, 54,66,67, 68,69, 70, 72,91,93, 94,96, 97, 106,111,112,118, 119, 140, 143, 147, 154,166, 174,175, 176,183,184,185,188,189, 191,195])
	Case Reports	([4])	([22,150, 198,200])
	Editorial	([1])	([170])
	Hypothesis	([1])	([199])
